# Legacy Mercury Re-emission and Subsurface Migration at Contaminated Sites Constrained by Hg Isotopes and Chemical Speciation

**DOI:** 10.1021/acs.est.3c07276

**Published:** 2024-03-12

**Authors:** Wei Zhu, Zhonggen Li, Ping Li, Jonas Sommar, Xuewu Fu, Xinbin Feng, Ben Yu, Wei Zhang, Ana T. Reis, Eduarda Pereira

**Affiliations:** † State Key Laboratory of Environmental Geochemistry, Institute of Geochemistry, Chinese Academy of Sciences, Guiyang 550081, China; ‡ Department of Forest Ecology and Management, Swedish University of Agricultural Sciences, Umeå SE-90183, Sweden; § School of Resources and Environment, Zunyi Normal College, Zunyi 563006, China; ∥ University of Chinese Academy of Sciences, Beijing 100049, China; ⊥ EPIUnit—Instituto de Saúde Pública, Universidade do Porto, Porto 4050-600, Portugal; # Laboratório para a Investigação Integrativa e Translacional em Saúde Populacional (ITR), Porto 4050-600, Portugal; ¶ LAQV-REQUIMTE—Associated Laboratory for Green Chemistry, University of Aveiro, Aveiro 3810-193, Portugal

**Keywords:** Hg contaminated sites, re-emission, subsurface migration, Hg isotope fractionation, Hg speciation, MIE, NVE

## Abstract

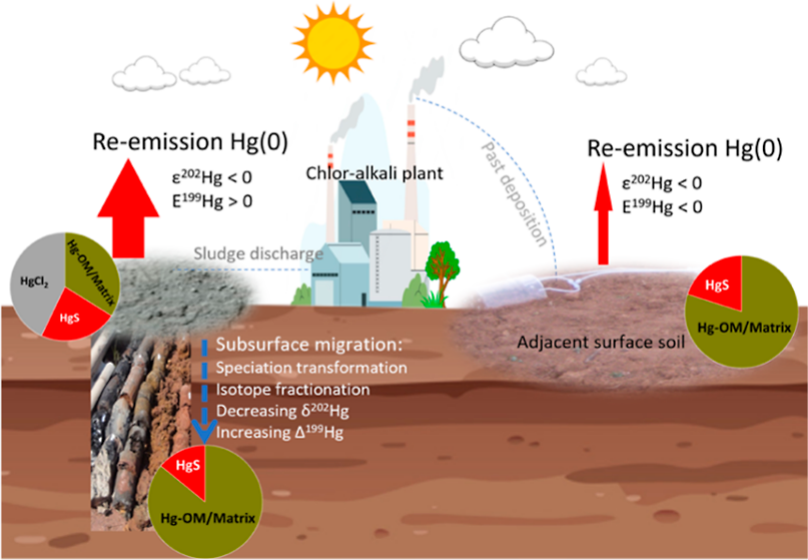

The re-emission and subsurface migration of legacy mercury (Hg) are not well understood due to limited knowledge of the driving processes. To investigate these processes at a decommissioned chlor-alkali plant, we used mercury stable isotopes and chemical speciation analysis. The isotopic composition of volatilized Hg(0) was lighter compared to the bulk total Hg (THg) pool in salt-sludge and adjacent surface soil with mean ε^202^Hg_Hg(0)-THg_ values of −3.29 and −2.35‰, respectively. Hg(0) exhibited dichotomous directions (*E*^199^Hg_Hg(0)-THg_ = 0.17 and −0.16‰) of mass-independent fractionation (MIF) depending on the substrate from which it was emitted. We suggest that the positive MIF enrichment during Hg(0) re-emission from salt-sludge was overall controlled by the photoreduction of Hg(II) primarily ligated by Cl^–^ and/or the evaporation of liquid Hg(0). In contrast, O-bonded Hg(II) species were more important in the adjacent surface soils. The migration of Hg from salt-sludge to subsurface soil associated with selective Hg(II) partitioning and speciation transformation resulted in deep soils depleted in heavy isotopes (δ^202^Hg = −2.5‰) and slightly enriched in odd isotopes (Δ^199^Hg = 0.1‰). When tracing sources using Hg isotopes, it is important to exercise caution, particularly when dealing with mobilized Hg, as this fraction represents only a small portion of the sources.

## Abstract 2

This study demonstrates a large extent of Hg isotope fractionation that overprinted the source signature during Hg(0) re-emission and subsurface Hg(II) migration at contaminated sites and has important implications for source and process tracing.

## 1 Introduction

Anthropogenic and natural sources release neurotoxic mercury (Hg) into the atmosphere, including the re-emission of legacy Hg.^1,2^ Once released, Hg can be dispersed globally *via* long-distance transport of atmospheric elemental Hg [Hg(0)].^3^ Since the preindustrial era, anthropogenic emissions have increased global soil and sediment Hg concentrations by a factor of three to four times.^4^ Contaminated sites, resulting from historical industrial and mining activities such as the chlor-alkali industry and artisanal and small-scale gold mining, can have dramatically higher levels of Hg in environmental media such as soils, air, and water, compared to background sites, sometimes by several orders of magnitude.^5−9^ However, legacy Hg at contaminated sites that lack confinement is prone to mobilize through re-emission into the atmosphere and migration into the hydrosphere. Although current global Hg inventories do not account for the contribution of Hg emission from historically contaminated sites,^2^ approximately 3000 identified contaminated sites were estimated to re-emit about 82 tons of Hg annually into the atmosphere and release about 116 tons into the hydrosphere.^10^ To reduce emissions and human exposure to Hg, the legally binding intergovernmental treaty, the *Minamata Convention on Mercury*, entered into force in 2017 (www.mercuryconvention.org). Aggressive actions to phase out intentional use of Hg and closure of existing point sources are expected to be implemented.^11^ This will lead to an increase in the number of legacy contaminated sites worldwide. To date, little is known about the processes driving the mobilization of legacy Hg from contaminated sites, making it difficult to predict the fate and transport of legacy Hg, incorporate it into the global Hg inventory, and assess the risk of legacy Hg to watersheds.^12^

For more than a century, the chlor-alkali industry has used the mercury-cell (Castner-Kellner) process as one of three manufacturing processes to produce chlorine gas and caustic soda. Due to the rapid phasing-out of this technology, annual global Hg(0) emissions from Hg cell chlor-alkali production have declined by 47% from 2010 (28.4 tons) to 2015 (15.2 tons).^12^ However, emissions of Hg to the atmosphere and release into the aquatic environment still occur from two central legacy sources: (1) historic solid waste disposal (*i.e.*, salt-sludge) and (2) the adjacent land surrounding the abandoned Hg cell perimeter.^13,14^ The initial deposited salt-sludge discharged from the electrolytic cell is dominated by Hg(0) and chloro-mercurates (*e.g.*, HgCl_3_^–^ and HgCl_4_^2–^) and therefore unconditionally of a mercurous chloride (Hg_2_Cl_2_) pool.^15^ The pristine surface soil adjacent to the chlor-alkali plant was contaminated by atmospheric Hg deposition,^14^ where the legacy Hg is primarily bound to organic matter or minerals.^5^

The stable Hg isotopes systematics, expressed as mass-dependent fractionation (MDF) and odd- and even mass-independent fractionation (MIF),^16^ are powerful tools for Hg source^17−19^ and process^7,20,21^ tracing. Hg isotope trajectories, including conventional MDF (typically reported as δ^202^Hg) and MIF (including odd-MIF Δ^199^Hg and Δ^201^Hg, even-MIF Δ^200^Hg), have been experimentally quantified for most environmentally relevant kinetic and equilibrium reactions.^22^ MDF occurs during physical, chemical, and biological processes, while odd-MIF is triggered only by the magnetic isotope effect (MIE) and nuclear volume effect (NVE), making it useful for tracking specific transformation processes. MIE arises during some photochemical reactions [*e.g.*, Hg(II)-O/N and Hg(II)-SR photoreduction],^23−25^ while NVE occurs during processes not requiring light [*e.g.*, organic matter-mediated Hg(II) reduction, equilibrium Hg(II)-thiol complexation, and Hg(0) vapor evaporation]^26−29^ and a share of light-induced reactions [*e.g.*, photoreduction of dissolved Hg(II) dominated by HgCl_2_ and HgC_2_O_4_].^30,31^ However, the magnitude and sign of MIF depend on the type of ligation [*e.g.*, Hg(II)-SR, Hg(II)-O/N, and Hg(II)-Cl] and the reaction mechanism,^25,30,31^ which makes tracing Hg transformation processes difficult. A particular challenge is posed by contaminated sites with Hg present in significant pools of different oxidation states and ligations. Combined chemical speciation and stable isotope studies have been successfully applied to track the subsurface Hg transformation processes at Hg(II) chloride-contaminated legacy industrial sites, where distinct Hg isotopic compositions observed in soil and groundwater matrices were linked to solid–liquid phase sorption and dark abiotic equilibrium redox reaction between Hg(II) and Hg(0).^7,21^

As little is known about the transformation processes that mobilize legacy Hg from contaminated sites, this study combines stable Hg isotope signatures and chemical speciation to address the mechanistic controls on (1) Hg(0) re-emission from the salt-sludge and adjacent natural surface soils and (2) subsurface migration of legacy Hg in the salt-sludge to soil continuum system. To address objective one, we determined the Hg(0) re-emission flux from salt-sludge and adjacent surface soils under controlled environmental conditions. We used chemical speciation and Hg isotope signatures to identify the mechanistic controls of Hg(0) re-emission. To achieve objective two, we determined the Hg chemical speciation and isotope signatures (in mobile and bulk pools) of three salt-sludge to soil continuum cores and one adjacent natural soil core. This allowed us to track the vertical migration of legacy Hg from heavily contaminated salt-sludge to subsurface soils.

## 2 Materials and Methods

### 2.1 Site Description and Sampling

In March 2012, samples were collected from a decommissioned chlor-alkali industrial plant (CIP) and a nearby natural land surface situated in Kunming city municipality, Southwest China (24.90°N, 102.46°E, 1850 m above sea level, Figure S1). The samples included three Hg contaminated salt-sludge to soil continuum cores, a reference natural soil core, and adjacent surface soils. The soils in the sampling area are typical well-drained red loam soil with low total organic carbon content (≤2%) containing a substantial amount of clay and sand-limestone fragments. The CIP produced chlorine, caustic soda, and polyvinyl-chloride (PVC) from 1962 to 1991 and 1971 to 2011, respectively. Liquid Hg(0) and mercuric chloride (HgCl_2_) were used in the chlor-alkali and PVC production facilities, respectively, resulting in environmental Hg contamination through waste dumping and atmospheric emission.^8^ After the industrial facility was closed, the major legacy reservoirs of Hg, which include the salt-sludge stockpile^32^ and the surrounding contaminated natural lands within a radius of approximately 6.5 km,^14^ were left in place. Salt-sludge heavily contaminated with Hg (0.43–2640 mg Hg kg^–1^) was discharged from liquid Hg(0) used in the electrolytic cell-room. The salt-sludge was piled directly on the soil surface in an area of approximately 1.1 Ha, which is surrounded by a concrete fence. The mobilization of Hg from the upper salt-sludge to subsurface soil raised groundwater Hg concentration up to 3.6 mg L^–1^.^32^ Previous publications have documented detailed information about the history of the site and its characteristics of Hg contamination.^8,14,32^ Three cores (SS-7, SS-8, and SS-22) were drilled with a 130 mm stainless-steel rig, extending to a subsurface depth of approximately 7 m. Each core consisted of an upper section of salt-sludge, with an average depth of 2.71 ± 0.67 m (1σ), and a lower section of natural soil (Figure S2). The sampling depth was approximately 7 m, which was above the groundwater level. The soil core REF-S was drilled in a *Prunus persica* orchard farmland located 2.1 km from the CIP.^14^ Additionally, surface soil samples from agricultural lands adjacent to the CIP were collected (refer to Figure S1). The soil and salt-sludge samples were stored in two layers of polyethylene bags and kept in the dark until laboratory Hg(0) re-emission experiments and chemical analysis.

### 2.2 Measurements of Hg(0) Re-emission Fluxes, Hg Speciation Analysis, and Ancillary Chemical Analysis

A single-pass gas exchange chamber (GEC) system (Figure S3) was used to mimic Hg(0) re-emission from two surface salt-sludge samples and five adjacent surface soils.^33,34^ The protocols for carrying out the Hg(0) re-emission experiments are detailed in Text S1. For the Hg(0) re-emission experiments, a total of 65 g of surface soil was used to place in quartz CEC (internal volume of 1.5 L). When using the high Hg(0) emitting salt-sludge as the substrate, the applied mass was limited to 6 g. Hg(0) was emitted from the substrates to the flushing 6.5 L min^–1^ Hg-free air (zero air) through the GEC under controlled environmental conditions [800 W m^–2^ solar irradiation (300–800 nm, light source were provided by an Oriel Solar Simulators, Newport, USA), 30 °C soil temperature and 15 wt % soil moisture]. Hg(0) exiting the GEC was collected onto a chlorine-impregnated activated carbon (ClC) trap for isotope analysis in the re-emission experiment. The trap consisted of approximately 1.0 g of ClC material filled in a 12 mm inner diameter and 100 mm long borosilicate glass tube.^35^ The flow rate during collection was 5.5 L min^–1^. A Tekran 2537B mercury vapor analyzer was used to measure the GEC inlet zero-air and outlet air Hg(0) concentration sequentially at a flow rate of 1.0 L min^–1^. To capture at least 20 ng of Hg on the ClC trap, the time required for each experiment varied between 1.2 and 15 h, depending on the magnitude of Hg(0) efflux. The sampled ClC-traps were stored in three-layer polyethylene bags and kept at 4 °C until analysis. Hg(0) flux was calculated as follows
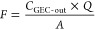
1where *F* is the Hg(0) emission flux (ng m^–2^ h^–1^), *A* is the substrate surface area (m^2^), *Q* is the flow rate (m^3^ h^–1^), and *C*_GEC-out_ represents average Hg(0) concentration in the GEC outlet gas (ng m^–3^).

The water-soluble Hg pool in the SS-7 and SS-22 core samples was extracted using 50 mL Falcon tubes. 5 g of the sample was mixed with 30 mL of Milli-Q water and left to equilibrate for approximately 12 h on a reciprocal shaker (50 rpm). The resulting slurry was then centrifuged at 3050*g* for 15 min, and the supernatant was filtered through a 0.45 μm Filtropur S Sarstedt filter. An aliquot of 0.2 M BrCl (0.5 vol %) was added to the filtered extracts to oxidize any dissolved Hg, and the solutions were stored at 4 °C until the analysis of Hg concentration and isotopic composition.

Chemical speciation of Hg in salt-sludge and soils was determined by thermo-desorption atomic absorption spectroscopy (TD-AAS) analysis.^36^ Samples were heated under increasing temperatures from 76 to 768 °C, and released Hg was measured using LECO AMA-254. The Hg chemical speciation was reconstructed by comparing its characteristic release curves with those of standard Hg compounds. The TD-AAS analysis was performed on air-dried samples. To assess the potential loss of Hg(0) during air-drying of samples (∼20 °C), we determined the differences in total Hg concentration (normalized to dry weight) between intact wet and air-dried samples.^37^ We also determined the total C and N contents in selected soils using an elemental analyzer (Vario MACRO Cube, Elementar).^20^

### 2.3 Hg Concentration and Stable Hg Isotopes Analysis

The Lumex RA-915+ Hg vapor analyzer coupled with a PYRO 915+ pyrolysis atomizer was used to determine the THg concentration in salt-sludge and soil samples.^38^ Solid samples (approximately 0.05–0.2 g) were extracted in 5 mL of freshly prepared aqua regia (1HNO_3_/3HCl, v/v).^14^ The sampled ClC-traps and REF-S core soils were processed using a double-stage oven combustion and acid trapping technique to release matrix-bound Hg into acid trapping solution (40%, v/v, 2HNO_3_/1HCl) for subsequent quantitative and isotopic analyses.^35,39^ Hg concentrations in acid-trapping solution, aqua regia extracts, and water-soluble extracts were determined on a cold-vapor atomic fluorescence spectrophotometer (Tekran 2500). The recovery of Hg(0) collected on the ClC-traps retrieved from oven-combustion and acid trapping yielded 88–96% recovery of online Tekran 2537B measurements (Text S1). The blanks of ClC (0.2 ± 0.1 ng Hg g^–1^, *n* = 12) accounted for <1.0% of Hg(0) collected on ClC-traps and were regarded as negligible.

The extraction and trapping solutions were diluted to a concentration of 1.0 ng Hg/mL (in approximately 20% vol of trapping-acid or 10% vol of aqua regia for consistency in respective analytical sessions) before analyzing Hg isotope ratios using MC-ICP-MS (Nu Plasma, Nu Instruments, UK).^40^ The Hg isotope signatures for MDF were reported in delta notation (δ) with δ^202^Hg (relative to the NIST-SRM-3133 bracketing standard)^16^
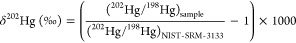
2MIF signature is calculated as
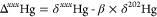
3where *xxx* represents mass numbers of 199, 200, and 201 with conversion factor β of 0.2520, 0.5024, and 0.7520, respectively. The uncertainty (2σ) of the reported Hg isotope signature was determined by measuring the reproducibility of the secondary standard UM-Almadén in respective sessions. Processing of UM-Almadén yielded isotopic values (δ^202^Hg = −0.57 ± 0.12‰, Δ^199^Hg = −0.02 ± 0.05‰, Δ^200^Hg = 0.01 ± 0.04‰, Δ^201^Hg = −0.03 ± 0.05‰, 2σ, *n* = 27) comparable with those presented in literature.^16^

Since the re-emission of Hg(0) accounted for less than 0.2‰ of the total Hg in the contaminated substrates (Section 3.2), the isotopic compositions of re-emission Hg(0) relative to the substrate total Hg can be approximated as apparent MDF enrichment factor (ε^202^Hg_Hg(0)-THg_) and MIF enrichment factors (*E*^199^Hg_Hg(0)-THg_, *E*^200^Hg_Hg(0)-THg_, and *E*^201^Hg_Hg(0)-THg_), respectively^41,42^
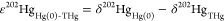
4
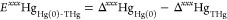
5

## 3 Results and Discussion

### 3.1 Hg Concentrations and Chemical Speciation

**Figure 1:**
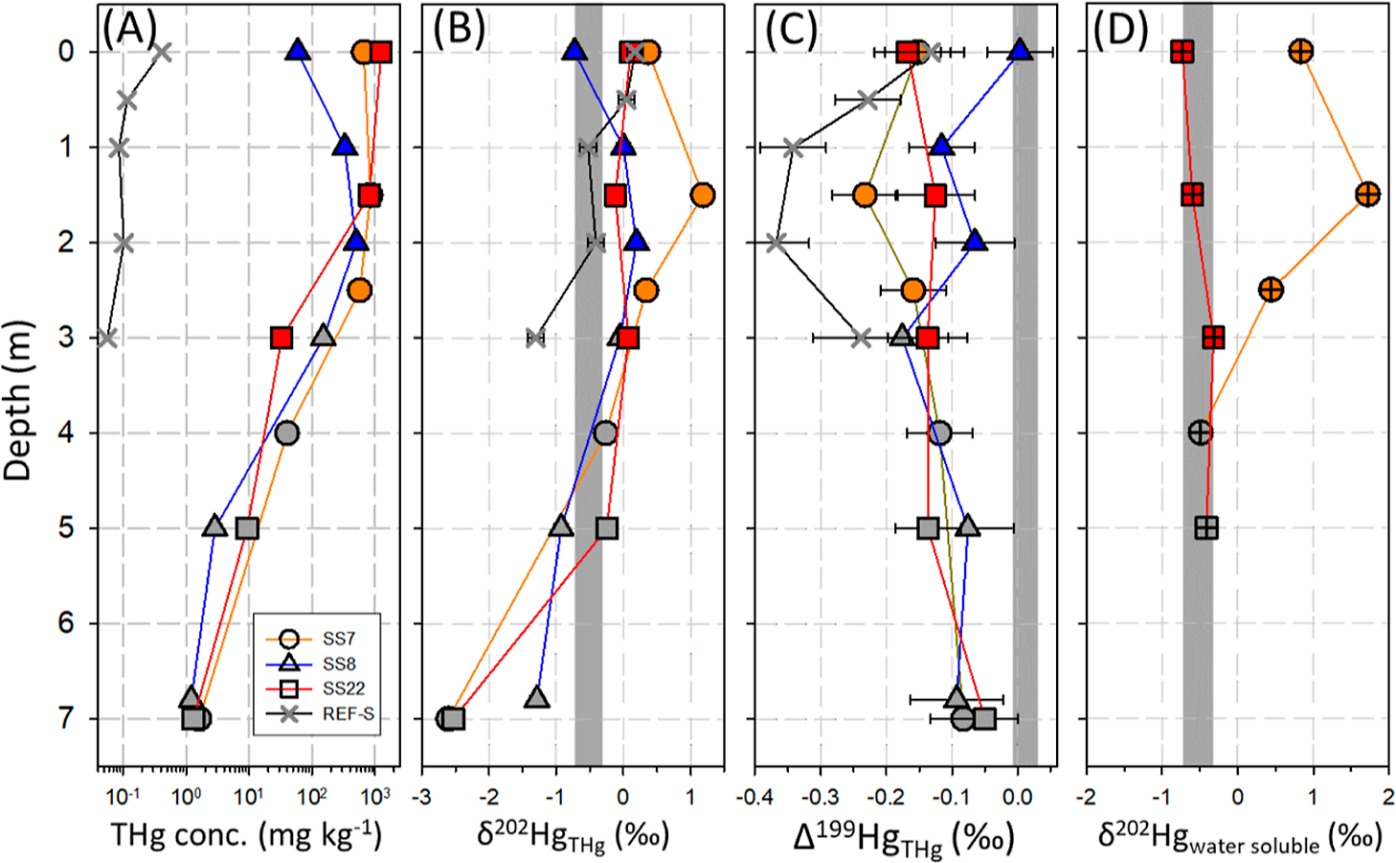
Total Hg (THg) concentration (A), isotope signatures of THg δ^202^Hg (B) and THg Δ^199^Hg (C), and water-soluble Hg δ^202^Hg (D) of salt-sludge (colored symbols) and soil (gray symbols) samples along three sludge–soil continuum cores (SS7, SS8, and SS22) and the REF-S adjacent natural soil core. The gray band in each subfigure represents the estimated Hg isotope signatures of the original liquid Hg(0) from the Wanshan Hg mine (cf. Section 3.3 and Text S2). Error bars represent ±2σ uncertainty values.

The geogenic background Hg recorded in the bottom soils of the REF-S profile (Figure 1a) was 0.05 mg kg^–1^. THg content in the five adjacent soils (0.3–4.8 mg kg^–1^, A1–A5, Table S1) surrounding the CIP was between 1 and 2 orders of magnitude higher than the geogenic background Hg.^14^ The long-term dispersion of the CIP emissions is also evident from the gradual increase in Hg concentrations in the REF-S profile from the deep to the upper surface soils. The concentration of THg in the three depth profiles of salt-sludge was higher (33.1–1246 mg kg^–1^) than that in the corresponding subsurface soils underneath (1.2–151 mg kg^–1^) (ANOVA, *p* < 0.01, Table S1). There was no vertical trend of total Hg in the salt-sludge. The significant variation of Hg in the salt-sludge suggests that the discharge from the electrolytic cell-room was initially heterogeneous, and/or there were potential postdumping losses of Hg, such as re-emission to the atmosphere and leaching to the subsurface layer. Figure 1a shows an apparent decreasing trend of total Hg in soils beneath the salt-sludge along the depth profiles, indicating the downward migration of Hg from the salt-sludge to the underlying soils. Even in the soil strata at ∼6.8–7.0 m with the lowest Hg content (1.2–1.6 mg kg^–1^), the downward flux contributed to >96% while geogenic Hg (0.05 mg kg^–1^) accounted for ≤4% of THg in the soil.

**Figure 2:**
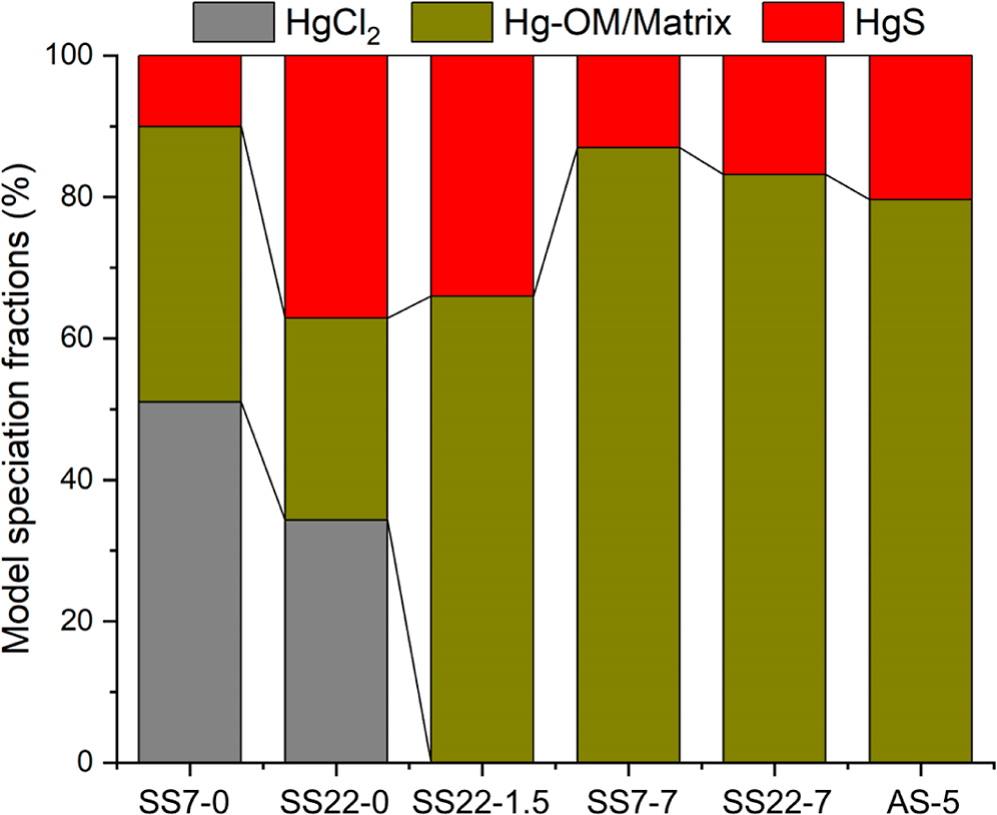
Fractions of Hg speciation (percentage of total Hg, %) in salt-sludge (SS7-0, SS22-0, and SS22-1.5), underneath (SS7-7 and SS22-7), and adjacent surface contaminated soils (AS-5) determined by TD-AAS. Corresponding TD-AAS spectra and peaks deconvolution are presented in Figures S4–S6. Note the coding of the samples from sludge-soil continuum cores SS*a*-*b* [*a* and *b* refer to the core number and sample depth (*m*), respectively].

The TD-AAS spectrum of Hg in salt-sludge and soils showed characteristic Hg-releasing curves in adjacent surface soils (AS-5, Figure S4), salt-sludge (SS7-0, SS22-0, SS22-1.5, Figure S5), and its subsurface soils (SS7-7, SS22-7, Figure S6). All samples released two major arrays of Hg forms: one desorbing at ∼250 °C and another desorbing at ∼300 °C. Given the low organic matter content in the salt-sludge and red loam soil (OC ≤ 2%, Table S1), we interpret these two forms to be matrix-bond Hg(II) [*i.e.*, Hg(II) bonded on organic matter and/or adsorbed on minerals]^6,7,37^ and precipitated solid phase HgS,^43^ respectively. Additionally, a Hg peak was detected at 160–180 °C in surface salt-sludge samples (SS7-0 and SS22-0), which matched the thermo-desorption curve of HgCl_2_ and Hg_2_Cl_2_ standards.^37,44^ Thus, we interpret this entity as mercuric and mercurous chloro complexes (*e.g.*, HgCl_2_, Hg_2_Cl_2_, HgCl_3_^–^, and HgCl_4_^2–^).^15^ The surface salt-sludge contains significant amounts (34–52%) of the chloro species mentioned earlier, as indicated by the Gaussian deconvolution of the TD-AAS spectra. This is followed by a change in Hg speciation to matrix-bond Hg(II) (66–89%) in the subsurface salt-sludge and soils along the profiles (Figure 2). In line with previous Hg speciation analyses of soils heavily impacted by deposition from chlor-alkali plants,^6,45^ the adjacent surface soils at our site were found to be dominated by ∼80% of matrix-bond Hg(II) and ∼20% of HgS (Figures 2 and S4).

The TD-AAS analysis was performed on air-dried samples stored in the dark at room temperature. A small amount of elemental Hg [Hg(0)] was detected, but it accounted for less than 0.4 and 0.1% of total Hg released from salt-sludge and its underlying soils at temperatures below 100 °C, where Hg(0) desorbs (Figures S4–S6). However, the air-drying process may lead to an underestimation of Hg(0) concentration due to its high volatility.^37^ To limit the potential significance of Hg(0) in salt-sludge and subsurface soils, we independently determined the total Hg concentration in the wet intact and air-dried samples along the depth profiles. The two methods produced highly consistent results (slope = 0.94, *R*^2^ = 0.99, Figure S7). Hg(0) may be a minor component of THg (≤6%), but it cannot be ruled out as a potential Hg species in the salt-sludge and subsurface soils.

### 3.2 Hg(0) Re-emission from Contaminated Sites and Isotopic Fractionation

The re-emission flux of Hg(0) from contaminated adjacent surface soils (A1–A5) ranged from 121 to 430 ng m^–2^ h^–1^ (Table S2). This value is 2 orders of magnitude higher than that of global background soil surfaces, which range from near-zero to a few ng m^–2^ h^–1^.^46−49^ Previous *in situ* investigations of Hg(0) emission from A1–A5 have shown that daytime flux (95–620 ng m^–2^ h^–1^) exceeded nighttime flux (8.7–55 ng m^–2^ h^–1^) by an order of magnitude.^14^ The re-emission flux of Hg(0) from the GEC system and the *in situ* daytime flux were comparable, indicating that the observed Hg(0) re-emission from the GEC system was primarily controlled by the photochemical reduction of soil legacy Hg. Not surprisingly, Hg(0) emissions from the salt-sludge were up to 3 orders of magnitude higher than those from adjacent soils (Table S2). The Hg(0) re-emission experiments were conducted under identical environmental conditions, including solar irradiation, temperature, and moisture. The large discrepancy in Hg(0) re-emission flux among the investigated samples is therefore largely due to the substrate characteristics.^33,50^ A linear correlation between soil THg concentration and Hg(0) re-emission flux (*R*^2^ = 0.88, *p* = 0.02, Figure S8) indicates that the THg concentration explains most of the variance of the Hg(0) re-emission flux. The regression model derived from the adjacent surface soils was used to extrapolate the Hg concentration levels of surface salt-sludge. The resulting factor of 2.0–5.3 times higher measured Hg(0) emission from the salt-sludge than the regression model predicts (Figure S8) suggests an intensified Hg(0) emission potential of salt-sludge compared with adjacent surface soils. This difference is likely due to the contrast in the Hg chemical speciation (Figure 2). Due to the significant amount of chloride-bound Hg(II)/Hg(I) in the salt-sludge (34–52%), Hg(II) has a much higher solubility (1–3%, Table S3) there than that in soils (more than 2 orders of magnitude). This provides a greater amount of available Hg for the chemical reactions. Furthermore, the complexation of Hg(II)/Hg(I) to Cl^–^ is weaker (log *k* = 6.7–15.2 for the chloro-mercurates)^51^ compared to that between Hg(II) and SR ligands (log *k* = ∼ 40.0 for Hg(OM-RS)_2_),^52^ which suggests a lower thermodynamic constraint in the formation of Hg(0) in salt-sludge than that in soils.

**Figure 3:**
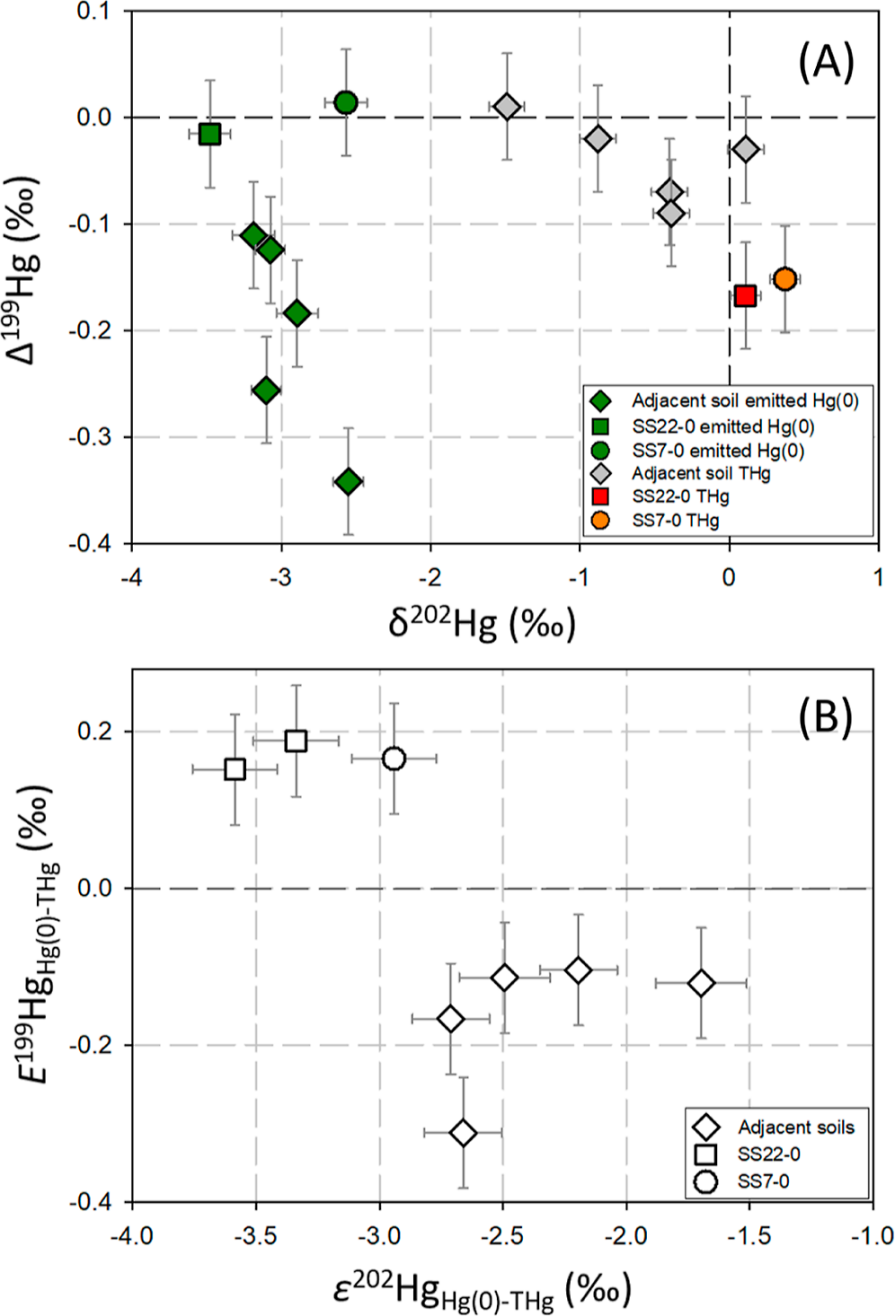
(A) δ^202^Hg *vs* Δ^199^Hg of re-emission Hg(0) from adjacent surface soils and surface salt-sludge and corresponding bulk total Hg; (B) Hg isotope enrichment factors ε^202^Hg_Hg(0)-THg_*vs E*^199^Hg_Hg(0)-THg_. The error bars represent the ±2SD analytical uncertainties (A) and propagated uncertainties (B), respectively. Note the coding of samples from sludge-soil continuum profiles SS*a*-*b* (*a* and *b* refer to profile number and sample depth [*m*], respectively).

The isotope compositions of Hg(0) re-emission from the adjacent surface soils were consistently characterized by negative δ^202^Hg (−3.19 to −2.55‰, *n* = 5) and Δ^199^Hg (−0.34 to −0.11‰, *n* = 5) signatures. While Hg(0) re-emission from the surface salt-sludge exhibited negative δ^202^Hg (mean = −3.09‰, *n* = 3) and near-zero Δ^199^Hg (mean = 0.01‰, *n* = 3) values (Figure 3a and Table S2). The calculated ε^202^Hg_Hg(0)-THg_ showed significant negative values from the adjacent surface soils (mean = −2.35‰, *n* = 5) and the surface salt-sludge (mean = −3.29‰, *n* = 3). On the other hand, *E*^199^Hg_Hg(0)-THg_ showed negative values for the adjacent surface soils (mean = −0.16‰, *n* = 5) but opposite positive values for the salt-sludge (mean = 0.17‰, *n* = 3), respectively (Figure 3b). The *E*^200^Hg_Hg(0)-THg_ was determined to be insignificant from zero (−0.01 ± 0.04‰ and 0.01 ± 0.01‰, 1σ, *n* = 5 and 3, *p* = 0.78 and 0.31 for adjacent surface soils and salt-sludge, respectively, as shown in Tables S1 and S2). This indicates the absence of even-isotope fractionation during Hg(0) re-emission from the terrestrial contaminated sites. It should be noted that the approximated isotopic enrichment factors may be challenged by the current unknown isotopic composition of photoreducible Hg(II) due to the potentially slightly unequal distribution of Hg isotopes among different Hg(II) species in contaminated substrates.^7^ Future studies are needed to better constrain the magnitude of isotope fractionation during Hg(0) re-emission from contaminated substrates.

**Figure 4:**
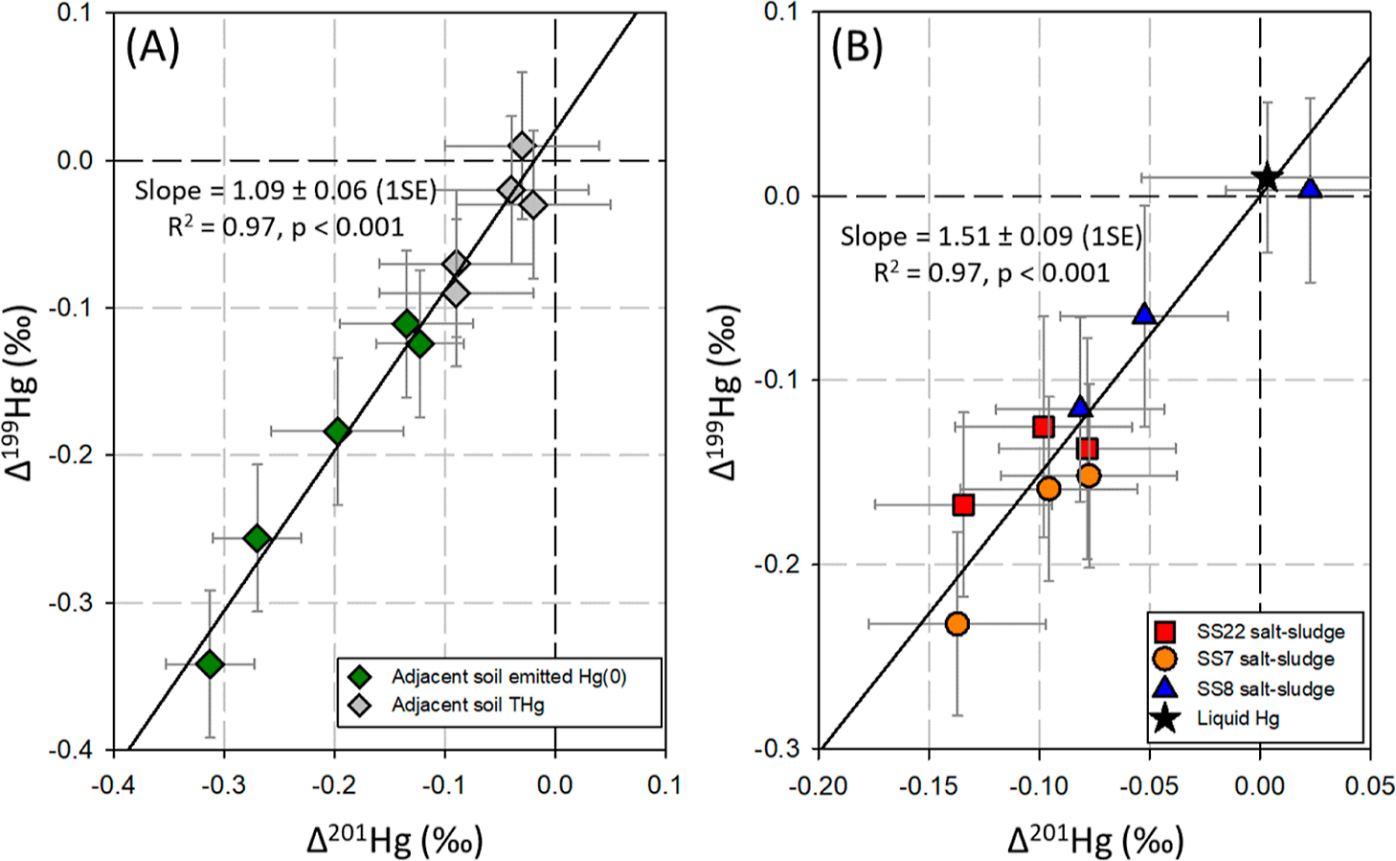
Scatter-plot of Δ^199^Hg versus Δ^201^Hg in (A) adjacent soil bulk total Hg and corresponding re-emission Hg(0) and (B) salt-sludge bulk total Hg.

The determined negative ε^202^Hg_Hg(0)-THg_ (mean = −2.35 and −3.29‰ for adjacent surface soils and salt-sludge, respectively) was generally larger than the experimental reported fractionation factors of photolytic (−1.8 to −0.6‰),^23−25,53^ nonphotolytic (−2.0 to −1.3‰),^23,26^ and biotic (−1.9 to −0.4‰)^54,55^ aqueous Hg(II) reduction, as well as Hg(0) evaporation/diffusion in aqueous and gaseous phases (−1.3 to −0.5‰).^56,57^ The abovementioned processes are closely related to the Hg(0) re-emission from the surface substrate to the atmosphere.^42^ These results highlighted that the MDF during Hg(0) re-emission from contaminated adjacent surface soils and salt-sludge is largely controlled by kinetic fractionation, which enriches light isotopes in the re-emission Hg(0). The negative *E*^199^Hg_Hg(0)-THg_ (mean = −0.16‰, *n* = 5) of Hg(0) observed during Hg(0) re-emission from adjacent soils is consistent with the negative MIF enrichment observed during Hg(0) emission reported from background agricultural soils (*E*^199^Hg_Hg(0)-THg_ = −0.27 to −0.13‰).^42^ The adjacent soil contains primarily Hg(II)-SR and HgS complexes (Figure 2). The observed re-emissions of Hg(0) are likely driven by photoreduction (Section 3.1). However, the small negative *E*^199^Hg_Hg(0)-THg_ is opposite to the positive MIF enrichment observed in experimental photolysis of sulfur-bonded Hg(II).^25^ In addition to the negatively directed enrichment of *E*^199^Hg_Hg(0)-THg_ versus ε^202^Hg_Hg(0)-THg_, the linear fit of the Δ^199^Hg/Δ^201^Hg data yielded a slope of 1.09 ± 0.06 (1SE, *p* < 0.001, Figure 4A), clearly indicating an underlying MIE mechanism responsible for Hg(0) efflux from soils. Our result demonstrated the overall negative MIF enrichment in Hg(0) re-emission from the adjacent surface soils resulted from synthetic effects of (+)MIE and (−)MIE during photolysis of Hg(II)-OR and Hg(II)-SR species, which is essentially consistent with photoreduction-driven Hg(0) volatilization from natural water^25^ and agricultural background soils and geogenic Hg-enriched soils.^41,42^ Although sulfur and/or matrix-bound Hg(II) species dominate in the adjacent surface soils (over 99%, as shown in Figure 2), the reduction of Hg(II)-OR is much faster (with reduction rates 3 orders of magnitude higher) than that of Hg-SR complexes.^58^ As a result, there is a slightly overall negative *E*^199^Hg_Hg(0)-THg_. Therefore, the Hg(0) re-emission from the contaminated substrates resulted from an array of processes including the reduction of different Hg(II) species and subsequent Hg(0) evaporation/diffusion from the matrix to the atmosphere, which may explain the more negative ε^202^Hg_Hg(0)-THg_ values than the abovementioned fractionation factors of photochemical and nonphotochemical Hg(II) reduction processes (−2.0 to −0.4‰).^23−26,53−55^

In contrast, the salt-sludge exhibited an enrichment of small positive odd-MIF (*E*^199^Hg_Hg(0)-THg_ = 0.17‰, *n* = 3) in Hg(0) re-emission, which was in the opposite direction to ε^202^Hg_Hg(0)-THg_ (mean = −3.29‰). The magnitude of *E*^199^Hg_Hg(0)-THg_ is similar to the odd-MIF anomaly induced by NVE during processes such as Hg(0) evaporation from liquid Hg(0),^28,29^ nonphotochemical Hg(II) reduction by natural organic matter (NOM),^26^ and indirect (secondary) photolysis of Hg(II)-Cl bound species.^30^ The small positive *E*^199^Hg_Hg(0)-THg_ during Hg(0) re-emission from salt-sludge is interpreted as being driven by indirect photolysis of Hg(II)-Cl complexes (*e.g.*, HgCl_2_) and/or Hg(0) evaporation from liquid/colloidal Hg(0) due to NVE. This interpretation is strongly supported by following multiple lines of evidence jointly. First, the extreme Hg(0) emission flux from the surface salt-sludge can only be explained by indirect photoreduction of less thermodynamically stable Hg(II)/Hg(I)-Cl complexes (which accounted for 34–52% of the bulk THg in the surface salt-sludge) and/or liquid Hg(0) evaporation. Second, Hg(0) re-emission from the salt-sludge resulted in Δ^199^Hg/Δ^201^Hg slope of 1.62 (±0.63, 1SE, Figure S9A), which is approximate to the diagnostic slope resulting from NVE-induced MIF anomalies (∼1.6).^26^ Third, the Δ^199^Hg/δ^202^Hg slope of −0.05 (±0.01, 1SE, Figure S9B) falls within the range reported for NVE-dominated isotope fractionation (Δ^199^Hg/δ^202^Hg = −0.36 to −0.01), such as Hg(0) evaporation from liquid Hg(0), dark abiotic Hg(0) oxidation, Hg(II) reduction by NOM, and indirect photolysis of HgCl_*x*_^2–*x*^ complexes.^24,26,28−30,59,60^ Fourth, the positive MIF in the re-emission Hg(0), along with the complementary negative MIF observed in the salt-sludge residual Hg(II), with a Δ^199^Hg/Δ^201^Hg slope of 1.51, indicates a NVE mechanism (see Section 3.3, Figure 4B). Notably, the mean ε^202^Hg_Hg(0)-THg_ value obtained from the salt-sludge was up to −0.91‰ more negative than that of the adjacent surface soil. As previously mentioned in Section 3.1, a small amount of liquid or colloidal Hg(0) in the salt-sludge may contribute to the occurrence of Hg speciation in the salt-sludge. The evaporation of Hg(0) from liquid or colloidal Hg(0) can generate a much greater magnitude of MDF enrichment due to its larger fractionation factor (α^202/198^ = 1.0067).^28^ Therefore, we interpret the much more negative ε^202^Hg_Hg(0)-THg_ from the salt-sludge (mean = −3.29‰) as being driven by significant re-emission of Hg(0) through evaporation from liquid or colloidal Hg(0).

The opposite direction of Δ^199^Hg enrichment in the Hg(0) emission from adjacent soils and salt-sludge highlights that the overall MIF values of Hg(0) re-emission from contaminated land surfaces are primarily determined by chemical forms of legacy Hg in the substrates. This is due to two reasons: (1) the trajectory of isotopic fractionation depends on Hg(II) speciation and (2) the rates of Hg(II) reduction are species-specific and kinetically constrained.

### 3.3 Hg Migration and Isotopic Fractionation in Salt-Sludge to Soil Continuum

The source isotopic signature of the original liquid Hg(0) could not be characterized as the liquid Hg(0) electrolysis technique has been phased out of the CIP for over two decades. It is known that the original liquid Hg(0) was produced by retorting cinnabar ores from the Wanshan Hg mine in Southwestern China (δ^202^Hg = −0.74 ± 0.11‰, Δ^199^Hg = 0.01 ± 0.02‰, Δ^200^Hg = 0.02 ± 0.04‰, 1σ, *n* = 13).^18^ At Wanshan Hg mine, the Hg isotope composition in calcine was reported (δ^202^Hg = 0.08 ± 0.20‰, Δ^199^Hg = 0.00 ± 0.02‰, 1σ, *n* = 11),^18^ and the Hg pools in calcine were found to be approximately 0.5%. Additionally, during the retorting of cinnabar at the Wanshan Hg mine, emitted Hg(0) was measured to be 10% on average (range: 2–32%),^61^ as previously documented. Using an isotopic mass balance method^62,63^ detailed in Text S2, we estimated that the exported liquid Hg(0) from the Wanshan Hg mine exhibited slightly heavier isotopes (mean δ^202^Hg = −0.60‰, range: −0.72 to −0.31‰) with similar MIF (mean Δ^199^Hg = 0.01‰) values of cinnabar. Only one salt-sludge sample (SS8-0, δ^202^Hg = −0.73‰, and Δ^199^Hg = 0.00‰) had a comparable isotopic signature to the original liquid Hg(0) (Table S1). The δ^202^Hg values of the bulk salt-sludge THg were consistently heavier than the liquid Hg(0) source signature, with a range from −0.73 to 1.18‰ (mean = 0.16 ± 0.50‰, 1σ, *n* = 9, Figure 1B). The bulk Δ^199^Hg values of the salt-sludge varied in a narrow range (−0.23 to 0.00‰) but showed significant negative enrichment (mean = −0.13 ± 0.07‰, 1σ, *n* = 9, *p* < 0.001) (Figure 1C). These results suggest that the isotopic composition of salt-sludge bulk Hg was indeed shifted from the original liquid Hg(0) source signatures through isotopic fractionation.

The linear regression analysis of isotopes in bulk salt-sludge THg resulted in slopes of 1.51 ± 0.09 (1SE) for Δ^199^Hg *vs* Δ^201^Hg and −0.12 ± 0.03 (1SE) for Δ^199^Hg *vs* δ^202^Hg (Figures 4B and S10, respectively). The negative MIF anomaly is consistent with NVE-dominated isotope fractionation (Δ^199^Hg/δ^202^Hg = −0.36 to −0.01), which can occur through processes such as dark abiotic Hg(0) oxidation,^59^ Hg(II) reduction by NOM,^26^ Hg(0) evaporation from liquid Hg(0),^28,29^ Hg(II)-thiol complexation,^27^ and photoreduction of mercuric species ligated by inorganic and organic ligands.^30,31^ The enrichment of heavy Hg isotopes in the salt-sludge is larger than that in the original liquid Hg(0) [mean Δ-δ^202^Hg_(salt-sludge)-(liquid Hg(0))_ = 0.76‰]. This is consistent with historical isotopic fractionation that preferentially yielded losses of lighter isotopes (*e.g.*, evaporation) from the salt-sludge. However, the poor correlation between the bulk THg concentration in salt sludge and δ^202^Hg (*R*^2^ = 0.22, *p* = 0.20, not shown, Figure S11) suggests that the significant variability in δ^202^Hg cannot be solely attributed to Hg loss, given the likely homogenized source signature of liquid Hg(0). The isotopic signatures of bulk salt-sludge THg may be influenced by fractionation during various processes. Initially, the liquid Hg(0) was recycled in the cell compartments^64^ during the chlor-alkali process, and the Hg pool was separated into precipitated salt-sludge that was discharged from the electrolysis cell-room. However, the salt-sludge bulk Hg pool represented only a small fraction of liquid Hg(0), providing ample opportunities to alter its isotopic signatures. Sediments contaminated by Hg wastes from four Swedish chlor-alkali plants, which used the same liquid mercury (δ^202^Hg = −0.5‰), were found to have a large variation in δ^202^Hg, ranging from −2.1 to 0.6‰. This variation was largely attributed to the chlor-alkali process.^64^ Second, the partial evaporation of liquid Hg(0) can cause a positive shift in δ^202^Hg and a slight negative Δ^199^Hg enrichment in the remaining Hg pool due to NVE.^28,29^ This is consistent with the MIF signatures found in the salt-sludge (mean = −0.13‰). Third, chemical speciation transformation in the salt-sludge followed by mobilization of Hg from the salt-sludge [*e.g.*, Hg(0) loss to the atmosphere and Hg(II) leaching to subsurface soils and aquifers], leaving residual Hg in the salt-sludge partitioned. Abiotic oxidation of the liquid Hg(0) to Hg(II) results in a positive MDF (ε^202^Hg = 1.54‰, due to the equilibrium isotope effect) and a small negative MIF (E^199^Hg = −0.18‰, due to NVE) in the Hg(II) product.^59^ This is in excellent agreement with the isotope enrichment in bulk salt-sludge Hg. Since Hg(II) species dominate the total Hg in present-day salt-sludge (Figure 2), it is highly plausible that the reduction of Hg(II) to Hg(0), followed by its subsequent loss to the atmosphere, occurs. The isotope enrichment during photoreduction-induced Hg(0) re-emission from salt sludge (ε^202^Hg_Hg(0)-THg_ = −3.29‰, E^199^Hg_Hg(0)-THg_ = 0.17‰, *n* = 3) is in the opposite direction of the respective positive MDF and negative MIF in bulk salt-sludge THg. This provides strong support for the observed anomalies of Hg isotope signatures in the salt-sludge. Additionally, the migration of Hg from the salt-sludge to the deeper soil layers resulted in the enrichment of light mercury isotopes in the underlying soils (Figure 1B, for further discussion see below). This may partially explain the observed enrichment of heavier mercury isotopes in the salt-sludge.

THg in soils located in the lower part of the sludge-soil continuum profiles showed significantly more negative δ^202^Hg values and slightly more positive Δ^199^Hg values compared to those of the salt-sludge (Figure 1). The δ^202^Hg values ranged from −2.61 to −0.05‰ (mean = −1.13 ± 1.07‰, 1σ, *n* = 7), while Δ^199^Hg values varied in a narrow range from −0.18 to −0.05‰ (mean = −0.10 ± 0.04‰, 1σ, *n* = 7). A gradual decrease in the soil bulk THg concentration and δ^202^Hg was observed vertically, accompanied by an increase in Δ^199^Hg along the depth profiles (Figure 1). Notably, significant negative and positive correlations were found between soil bulk THg δ^202^Hg *vs* 1/[THg] (*R*^2^ = 0.72, *p* < 0.05, Figure S11), and Δ^199^Hg *vs* 1/[THg] (*R*^2^ = 0.63, *p* < 0.05, Figure S12), respectively. Due to the exogenous Hg contamination source contributing to >96% of THg in the soil (Section 3.1), the isotopic compositions of the soil bulk THg should closely resemble that of the exogenous Hg source in all samples from the sludge–soil continuum profiles as controlled by the pool size effect.^65^ This argument was corroborated by the fact that subsurface soils in the sludge–soil continuum profiles had more negative δ^202^Hg and positive Δ^199^Hg values compared to both the REF-S core subsurface soils (which are of geogenic source) and the salt-sludge profiles (Figure 1). Therefore, the observed δ^202^Hg *vs* 1/[THg] and Δ^199^Hg *vs* 1/[THg] trends cannot be explained by end-member mixing of two different Hg sources (*i.e.*, salt-sludge and geogenic sources) in the present study. However, it appears that the observed significant shift of δ^202^Hg and Δ^199^Hg toward negative and positive enrichment, respectively, in soil THg at the deeper depth was caused by isotopic fractionation during the vertical Hg migration. This process preferentially enriched light and odd isotopes in the bulk mineral soil matrix.

Salt-sludge and soil extracts provided solid evidence of Hg isotope fractionation during legacy Hg migration in the subsurface system (Figure 1D). The water-soluble Hg pool in the uppermost subsurface soils (SS7-4.0 and SS22-5.0) exhibited slightly lighter δ^202^Hg than the bulk THg (mean Δ-δ^202^Hg_water-soluble-THg_ = −0.20‰, *n* = 2, Figure S13). The salt-sludge water-soluble Hg pool displayed an irregular pattern with variability in enrichment from notably lighter to heavier than the bulk THg (Δ-δ^202^Hg_water-soluble THg_ = −0.84 to 0.54‰, mean = −0.10‰, *n* = 6). The water-soluble Hg pool in soils and sediments has been observed to vary in its solubility, with some studies reporting substantially lower levels,^7,66,67^ while others report similar^42,68^ or higher^7,19,21,69^ levels of δ^202^Hg signatures compared to the bulk THg. This variation is thought to result from the differential binding of Hg isotopes in different chemical forms of Hg in the substrates, as well as the kinetic and equilibrium processes that desorb Hg(II) from the matrix surface during extraction.^21,64^ However, the uppermost soils (*e.g.*, SS8-3.0) that were just beneath the salt-sludge exhibited similar δ^202^Hg and Δ^199^Hg signatures to the average isotopic compositions of salt-sludge bulk THg (*p* > 0.05, *n* = 9). This suggests that there was negligible isotopic fractionation during leaching of Hg from the salt-sludge. Therefore, what caused the substantial enrichment of light and odd isotopes (δ^202^Hg = −0.05 to −2.53‰, Δ^199^Hg = −0.18 to −0.05‰) from the uppermost soils to the deepest soils? During the dissolution and readsorption processes, kinetic reactions tend to enrich the light isotopes in the product Hg^65,70^ on the soil matrix. The small positive Δ^199^Hg shift may result from NVE, as indicated by the opposite directions of MDF and MIF. This is further supported by the linear fitted Δ^199^Hg/Δ^201^Hg slope of 1.42 (±0.26, 1SE, *p* < 0.05, *n* = 7, Figure S14), which falls within the NVE diagnostical range reported in the literature. In the subsurface soils, Hg species were mainly composed of matrix-bound (∼85%) and HgS (∼15%) (Figures 2 and S6). On the other hand, Hg leached from the salt-sludge was likely dominated by Hg(II)-Cl due to its high solubility. The high Hg-binding capacity of soil is expected to retain the highly soluble Hg leached from the upper salt-sludge by rapid adsorption to soil NOM, minerals, and/or inorganic reduced sulfur and subsequent transformation to soil matrix-bound Hg(II) and HgS.^71^ Although the isotope compositions of Hg-OM, Hg adsorbed on minerals, and HgS in soils cannot be determined, it is reasonable to assume that there is an unequal distribution of Hg isotopes due to varying bonding strengths. In equilibrium systems, Hg(II) binds to –SR, adsorbs to goethite, and precipitates to HgS with varying enrichment factors of −0.6, −0.4, and −0.6‰, respectively.^27,72,73^ The soil water-soluble Hg pool (SS7-4.0 and SS22-5.0), which is isotopically consistently lighter than the bulk total Hg (Δ-δ^202^Hg_water-soluble-THg_ = −0.20‰, *n* = 2, Figures 1D and S13), supports the gradual decrease of δ^202^Hg in soil profiles (Figure 1B). To explain the observed δ^202^Hg trend in the soil profiles, we hypothesize that preferential dissolution of a fraction of Hg that is isotopically lighter than the bulk THg, such as dissolution of NOM bound Hg, from the soil matrix dominates the mobilized Hg in the subsurface equilibrium soil-water system. In the soil column, dissolved NOM bound Hg(II) is more mobile than HgCl_2_, Hg(0), and HgS, and therefore likely dominates the downward mobilization of Hg species in soil profiles.^74^ Therefore, we propose that the observed Hg isotope pattern (Figure 1B,C) is a result of equilibrium isotope fractionation, specifically Hg(II) sorption to NOM and/or minerals, as well as Hg(II) exchange among them.^27,72^ It should be noted that other processes, such as the dark abiotic reduction of Hg(II), may also occur in the soil profiles. This has been frequently observed in contaminated subsurface soils^7,21,71^ and can cause a shift of Δ^199^Hg and δ^202^Hg toward negative and positive in the residual Hg(II),^26^ respectively. However, this is opposite the observed δ^202^Hg−Δ^199^Hg pattern in Figure 1B,C. Therefore, it is unlikely to be a major driving process in the fractionation of Hg isotopes during vertical migration in this system.

## 4 Environmental Implications

To support global efforts to reduce anthropogenic mercury emissions and to phase out intentional use of mercury under the UNEP Minamata Convention, it is crucial to improve our understanding of legacy mercury at contaminated sites. Our study revealed significant Hg isotope fractionation that altered the source signatures during both surface re-emission and subsurface migration of legacy Hg at chlor-alkali industry contaminated sites. The re-emitted Hg(0) induced by photochemistry was generally enriched in light isotopes with distinct MIF enrichment. We also showed that the Hg isotopic signatures of the re-emitted Hg(0) were influenced by the interplay of chemical speciation (−O, −SR, and −Cl ligands)-dependent Hg isotope enrichment factors and species-specific Hg(II) reduction rates. The migration of Hg(II) from salt-sludge to subsurface soils resulted in the enrichment of negative MDF and positive MIF in deep soils. This is likely due to equilibrium isotopic fractionation associated with selective Hg(II) partitioning, such as Hg(II) binding to organic matter and adsorption to minerals and/or exchange among them as well as speciation transformation (*e.g.*, precipitation of β-HgS) in the subsurface soil environment. These insights into the environmental processes of legacy Hg provided a solid foundation for developing watershed process models to assess Hg fate and determine future remediation strategies.

The mobilized Hg, whether through re-emission of Hg(0) or migration into the subsurface environment, typically represents a small fraction of the original Hg source. As a result, it is susceptible to changes in the source’s isotope signature. Our findings have significant implications for the use of stable Hg isotopes in source tracing. Caution should be exercised when mobilized Hg (such as gaseous Hg(0) or leachate) represents a small portion of the source. Consistent with prior research, the use of chemical speciation and Hg isotope analysis has proven to be effective in tracing geochemical processes.^7,21^ Advanced techniques, such as XAS spectroscopy, can improve the quantification of Hg chemical speciation and aid in resolving complex environmental systems in future studies.

## Supporting Information Available

Detailed information on maps and photographs of field sampling and Hg(0) re-emission experiments; calculation of liquid Hg(0) isotope composition; all Hg concentration, Hg(0) flux, and Hg isotope compositions data; TD-AAS spectra; and ancillary data analysis (PDF)
