# Supplementary material for: Legacy Mercury Re-emission and Subsurface Migration at Contaminated Sites Constrained by Hg Isotopes and Chemical Speciation
Source: Environ Sci Technol. 2024 Mar 12;58(12):5336–46. doi: 10.1021/acs.est.3c07276 (PMC10976890; doi:10.1021/acs.est.3c07276)
Supplement: Supplementary file 1 — es3c07276_si_001.pdf [file es3c07276_si_001.pdf]

Supporting Information

Legacy Mercury Re-emission and Subsurface Migration at Contaminated Sites Constrained by Hg Isotopes and Chemical Speciation

Wei Zhu [1,2,*], Zhonggen Li [1,3], Ping Li [1,4], Jonas Sommar [1], Xuewu Fu [1,4], Xinbin Feng [1,4,*], Ben Yu [1], Wei Zhang [1], Ana T. Reis [5,6], Eduarda Pereira [7]

[1] State Key Laboratory of Environmental Geochemistry, Institute of Geochemistry, Chinese Academy of Sciences, Guiyang 550081, China
[2] Department of Forest Ecology and Management, Swedish University of Agricultural Sciences, SE-90183 Umeå, Sweden
[3] School of Resources and Environment, Zunyi Normal College, Zunyi 563006, China
[4] University of Chinese Academy of Sciences, Beijing 100049, China
[5] EPIUnit—Instituto de Saúde Pública, Universidade do Porto, 4050-600 Porto, Portugal
[6] Laboratório para a Investigação Integrativa e Translacional em Saúde Populacional (ITR), 4050-600 Porto, Portugal
[7] LAQV-REQUIMTE—Associated Laboratory for Green Chemistry, University of Aveiro, 3810-193 Aveiro, Portugal

**** Corresponding Authors:***

Wei Zhu, Phone: +46 (0)76 9682807; E-mail: wei.zhu@slu.se

Xinbin Feng, Phone: +86 851 85895728; E-mail*:* fengxinbin@vip.skleg.cn

SI lists of contents: 20 pages

Text: S1 – S2 (Page 2 – 3)
Tables: S1 – S3 (Pages 4 – 5)
Figures: S1 – S14 (Pages 6 – 19)
References: (Page 20)

Text S1. Hg(0) re-emission experiments

Hg(0) re-emission from the adjacent soils and salt-sludge samples was performed under identical conditions (i.e., 850 W m^{-2}, 35°C soil temperature, and 15 wt. % soil moisture), which are comparable to the midday environmental variables *in situ* during the season of late spring to summer season.[1] The instrumental setup, adapted from previous studies,[2, 3] is shown in Figure S3. A quartz ***gas exchange chamber (GEC)*** [4] (height: 8.5 cm, diameter: 15.0 cm, internal volume: 1.5 L) was used. For each specific sample, soil (~65 g) or salt-sludge (~6 g) samples were placed in the GEC. The bottom of the GEC was placed on a flat quartz plate and subsequently sealed with silicone sealant. The inlet of the GEC was supplied with Hg-free air from a Tekran® 1100 zero air generator and an additional zero air canister at a constant flow rate. The outflow of the GEC was connected to a Tekran® 2537B Hg vapor analyzer (flow rate: 1.0 L min^{-1}) and a chlorine-impregnated activated carbon trap (CLC-trap) followed by a vacuum pump (flow rate: ~5.5 L min^{-1}). Therefore, the continuous gas flow flushing through the GEC was maintained at ~6.5 L min^{-1}.

The GEC inlet (Hg-free air) and outlet gas Hg(0) concentrations were measured sequentially by the Tekran® 2537B using a Tekran 1110 manifold (three-way automated magnetic switch). A vacuum pump flush at 1.0 L min^{-1} was connected to the Tekran 1110 manifold to maintain a continuous flow through the GEC during the sequential Hg(0) measurement by the Tekran® 2537B (pump 2 in Figure S3). A 0.45 μm Teflon membrane filter and a PFA tube filled with soda-lime to protect the sampling gold cartridges were installed upstream the Tekran® 2537B.

Solar irradiance was provided by a solar simulator (Oriel Research Lamp 68911, Oriel Instruments) equipped with a xenon lamp (300 W, 300-800 nm, ozone-free, Oriel lamp). Solar radiation was adjusted to 800 W m^{-2} and covered the entire GEC footprint (i.e., soil or salt-sludge surface inside the GEC). Soil temperature was maintained at 30°C with (1) a modified heating plate capable of heating the GEC and (2) preheated mercury-free gas in a 2.5 L Teflon gas reservoir installed in front of the GEC. Soil moisture of 15% was determined gravimetrically by spraying the substrate with Mill-Q water (Millipore, 18.0 MΩ).[5] Actual irradiance and soil temperature were monitored throughout the experiment by a portable weather station (HOBO U-30, Onset Corp., USA).

Text S2. Isotopic composition of liquid Hg(0) from the Wanshan Hg mining area

The original liquid Hg(0) was not available to characterize the source isotopic signature of source because the use of liquid Hg(0) was discontinued more than two decades ago. We adopted a mass balance approach [6, 7] to constrain the isotope composition of the liquid Hg(0). The original liquid Hg(0) was documented by roasting cinnabar ores from the Wanshan Hg mine. Thus, a mass balance of Hg isotopes can therefore be established:

$$f_{calcine} \cdot \delta^{202}Hg_{calcine} + f_{liquid\ Hg(0)} \cdot \delta^{202}Hg_{liquid\ Hg(0)} + f_{GEM} \cdot \delta^{202}Hg_{GEM} = \delta^{202}Hg_{cinnabar} \quad (1)$$

$$f_{calcine} + f_{liquid\ Hg(0)} + f_{GEM} = 1 \quad (2)$$

Where the $f_{calcine}$, $f_{liquid\ Hg(0)}$, f_{GEM} represent the fractions of calcine, liquid Hg(0), and evaporation lost GEM Hg pools, respectively, during the roasting of cinnabar ores. The $\delta^{202}Hg_{calcine}$, $\delta^{202}Hg_{liquid\ Hg(0)}$, $\delta^{202}Hg_{GEM}$ represent the corresponding MDF values. $\delta^{202}Hg_{cinnabar}$ represents the MDF of the cinnabar ores (mean ± 1σ = -0.74 ± 0.11‰).[8] The residual calcine Hg pool ($f_{calcine}$ = ~ 0.5%, $\delta^{202}Hg_{calcine}$ = 0.08 ± 0.20‰, 1σ) during cinnabar roasting at the Wanshan Hg mine accounted for only a small fraction of the total cinnabar Hg pool.[8] Given the low $f_{calcine}$, total Hg(0) (i.e., the sum of liquid Hg(0) and evaporative GEM lost) can be considered to preserve cinnabar Hg isotopes. Equations (1) and (2) can be simplified as

$$f_{liquid\ Hg(0)} \cdot \delta^{202}Hg_{liquid\ Hg(0)} + f_{GEM} \cdot \delta^{202}Hg_{GEM} = \delta^{202}Hg_{cinnabar} \quad (3)$$

$$f_{liquid\ Hg(0)} + f_{GEM} = 1 \quad (4)$$

Sun et al. (2016) demonstrated that liquid-vapor Hg(0) fractionation during cinnabar roasting can be assumed to be close to equilibrium Hg(0) evaporation from liquid Hg(0), and isotopic enrichment between liquid Hg(0) and evaporation lost GEM can be established:[7]

$$\delta^{202}Hg_{liquid\ Hg(0)} - \delta^{202}Hg_{GEM} = \varepsilon^{202}Hg_{liquid-vapor\ equilibrium} \quad (5)$$

Where the enrichment factor $\varepsilon^{202}Hg_{liquid\text{-}vapor\ equilibrium}$ = 1.1‰ (range: 0.9 to 1.4‰).[9, 10]

Using the mass balance dataset of Hg emission factors f_{GEM} (mean f_{GEM} = 10%, range: 2 to 32%) during cinnabar roasting in the Wanshan Hg mining area developed by Li et al., (2009),[11] and combining the eq.s (3) – (5), the $\delta^{202}Hg_{liquid\ Hg(0)}$ can be resolved (mean $\delta^{202}Hg_{liquid\ Hg(0)}$ = -0.60‰, range: -0.72 to -0.31). Similarly, the $\Delta^{199}Hg_{liquid\ Hg(0)}$ is calculated to be similar to the MIF value for cinnabar ore (mean = 0.01‰).

Table S1. The total Hg concentration and isotope signatures of the salt-sludge and soil samples. Note the coding of the samples from sludge-soil continuum cores SS*a*-*b* (*a* and *b* referred to core number and sample depth [m], respectively). The "OC" refers to organic carbon content.
* Note the total Hg concentration and isotopic signature of adjacent surface soils have previously been published in Zhu et al., 2018.[1]

Samples Label	Classfication	Description	Depth	THg	**δ^{202}Hg**	2σ	**Δ^{199}Hg**	2σ	Δ^{200}Hg	2σ	**Δ^{201}Hg**	2σ	OC
			(m)	(mg/kg)	**(‰)**	(‰)	**(‰)**	(‰)	(‰)	(‰)	(‰)	(‰)	%
SS7-0	SS7 core	Salt-sludge	0.0	663	**0.37**	0.10	**-0.15**	0.05	0.01	0.04	**-0.08**	0.04	
SS7-1.5		Salt-sludge	1.5	847	**1.18**	0.10	**-0.23**	0.05	-0.02	0.04	**-0.14**	0.04	
SS7-2.5		Salt-sludge	2.5	574	**0.34**	0.10	**-0.16**	0.05	0.01	0.04	**-0.10**	0.04	
SS7-4.0		Soil	4.0	40.3	**-0.26**	0.10	**-0.12**	0.05	-0.01	0.04	**-0.08**	0.04	0.9
SS7-7.0		Soil	7.0	1.62	**-2.61**	0.10	**-0.08**	0.05	0.01	0.04	**-0.05**	0.04	0.7
SS8-0	SS8 core	Salt-sludge	0.0	59.3	**-0.73**	0.10	**0.00**	0.05	-0.03	0.04	**0.02**	0.04	
SS8-1.0		Salt-sludge	1.0	331	**0.00**	0.10	**-0.12**	0.05	0.03	0.04	**-0.08**	0.04	
SS8-2.0		Salt-sludge	2.0	502	**0.19**	0.10	**-0.07**	0.06	0.04	0.04	**-0.05**	0.04	
SS8-3.0		Soil	3.0	151	**-0.05**	0.10	**-0.18**	0.07	-0.02	0.04	**-0.12**	0.04	
SS8-5.0		Soil	5.0	2.86	**-0.93**	0.10	**-0.08**	0.07	0.01	0.04	**-0.04**	0.04	
SS8-6.8		Soil	6.8	1.21	**-1.29**	0.10	**-0.09**	0.07	-0.04	0.04	**-0.08**	0.04	
SS22-0	SS22 core	Salt-sludge	0.0	1246	**0.11**	0.10	**-0.17**	0.05	0.01	0.04	**-0.13**	0.04	
SS22-1.5		Salt-sludge	1.5	811	**-0.12**	0.10	**-0.13**	0.06	-0.04	0.04	**-0.10**	0.04	
SS22-3.0		Salt-sludge	3.0	33.1	**0.07**	0.10	**-0.14**	0.06	0.02	0.04	**-0.08**	0.04	2.0
SS22-5.0		Soil	5.0	9.29	**-0.25**	0.10	**-0.14**	0.05	0.01	0.04	**-0.07**	0.04	1.0
SS22-7.0		Soil	7.0	1.31	**-2.53**	0.10	**-0.05**	0.05	0.03	0.04	**-0.03**	0.04	0.7
REF-S-0	Adjacent natural reference soil core	Soil	0.0	0.41	**0.17**	0.12	**-0.13**	0.05	0.01	0.04	**-0.13**	0.07	
REF-S-0.5		Soil	0.5	0.12	**0.05**	0.12	**-0.23**	0.05	-0.02	0.04	**-0.23**	0.07	
REF-S-1.0		Soil	1.0	0.08	**-0.53**	0.12	**-0.34**	0.05	-0.01	0.04	**-0.32**	0.07	
REF-S-2.0		Soil	2.0	0.10	**-0.41**	0.12	**-0.37**	0.05	-0.03	0.04	**-0.36**	0.07	
REF-S-3.0		Soil	3.0	0.05	**-1.31**	0.12	**-0.24**	0.07	0.04	0.01	**-0.17**	0.07	
AS-1	Adjacent surface soil*	Soil	0	0.31	**-1.49**	0.12	**0.01**	0.05	-0.02	0.04	**-0.03**	0.07	2.0
AS-2		Soil	0	0.69	**-0.40**	0.12	**-0.07**	0.05	0.00	0.04	**-0.09**	0.07	1.6
AS-3		Soil	0	0.55	**-0.88**	0.12	**-0.02**	0.05	0.00	0.04	**-0.04**	0.07	
AS-4		Soil	0	1.30	**-0.39**	0.12	**-0.09**	0.05	0.08	0.04	**-0.09**	0.07	2.7
AS-5		Soil	0	4.79	**0.11**	0.12	**-0.03**	0.05	0.00	0.04	**-0.02**	0.07	4.8

Table S2. Hg(0) flux and isotope signatures of Hg(0) emitted from soil and salt-sludge samples under simulated environmental conditions.

Substrate	Description	Hg(0) flux (ng m^{-2} h^{-1})		Fraction of emitted Hg(0)	**δ^{202}Hg**	2σ	**Δ^{199}Hg**	2σ	Δ^{200}Hg	2σ	**Δ^{201}Hg**	2σ
		Mean	1 SD	%	**‰**	‰	**‰**	‰	‰	‰	**‰**	‰
AS-1	Emitted Hg(0)	121	7.1	0.194	**-3.19**	0.14	**-0.11**	0.05	0.01	0.04	**-0.14**	0.06
AS-2	Emitted Hg(0)	204	19.7	0.114	**-2.89**	0.14	**-0.18**	0.05	-0.01	0.04	**-0.20**	0.06
AS-3	Emitted Hg(0)	103	9.8	0.115	**-3.07**	0.10	**-0.12**	0.05	0.02	0.04	**-0.12**	0.04
AS-4	Emitted Hg(0)	269	16.1	0.091	**-3.10**	0.10	**-0.26**	0.05	0.00	0.04	**-0.27**	0.04
AS-5	Emitted Hg(0)	430	39.0	0.021	**-2.55**	0.10	**-0.34**	0.05	0.01	0.04	**-0.31**	0.04
SS22-0	Emission Hg(0)	162292	14838	0.010	**-3.48**	0.14	**-0.02**	0.05	0.01	0.04	**-0.04**	0.06
SS22-0	Emission Hg(0)	187644	20314	0.007	**-3.23**	0.14	**0.02**	0.05	0.02	0.04	**-0.05**	0.06
SS7-0	Emission Hg(0)	228556	24420	0.012	**-2.57**	0.14	**0.01**	0.05	0.03	0.04	**0.03**	0.06

Table S3. The water-extracted Hg concentrations and isotope signatures of SS7 and SS22 cores.

Extraction samples	Description	Dissolved Hg	Fraction of substrate total Hg	**δ^{202}Hg**	2σ	**Δ^{199}Hg**	2σ	Δ^{200}Hg	2σ	**Δ^{201}Hg**	2σ
		μg L^{-1}	%	**‰**	‰	**‰**	‰	‰	‰	**‰**	‰
SS7-0	Water soluble Hg	3039	2.75	**0.74**	0.13	-0.12	0.05	-0.01	0.05	-0.06	0.05
SS7-1.5	Water soluble Hg	2379	1.69	**1.72**	0.13	-0.27	0.05	-0.01	0.05	-0.12	0.05
SS7-2.5	Water soluble Hg	1848	1.93	**0.44**	0.10	-0.12	0.06	-0.02	0.04	-0.07	0.04
SS7-4.0	Water soluble Hg	1.6	0.02	**-0.49**	0.10	-0.05	0.06	0.02	0.04	-0.08	0.04
SS22-0	Water soluble Hg	194	0.93	**-0.73**	0.10	-0.10	0.07	0.04	0.04	-0.07	0.04
SS22-1.5	Water soluble Hg	68	0.50	**-0.60**	0.10	-0.11	0.07	0.02	0.04	-0.05	0.04
SS22-3.0	Water soluble Hg	13.3	0.24	**-0.32**	0.10	-0.13	0.07	-0.01	0.04	-0.06	0.04
SS22-5.0	Water soluble Hg	1.1	0.07	**-0.41**	0.10	-0.16	0.07	-0.01	0.04	-0.12	0.04

Figure S1. Map showing the sampling locations of the CIP, five adjacent surface soils (AS1 - AS5), reference soil core (REF-S), and the location of salt-slurry stockpile.

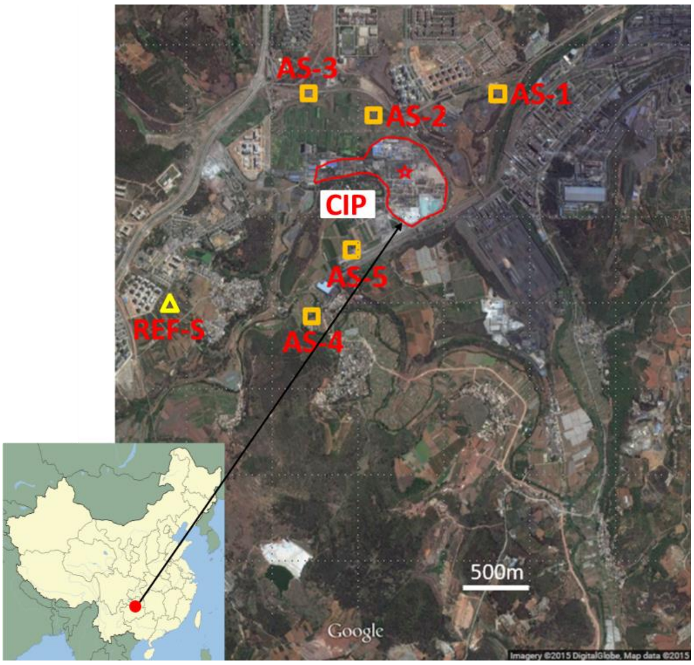

Figure S2. Photos shown: (A) a glance of the salt-slurry stockpile inside the CIP (cf. Figure S1); (B) schematic contour of the salt-slurry stockpile and spatial distribution of three salt-sludge – soils continuum cores (SS7, SS8 and SS22) (modified from Wang et al., 2019 [12]); (C) photo of mechanically drilling of the salt-slurry – soil cores; (D) a salt-slurry – soil core.

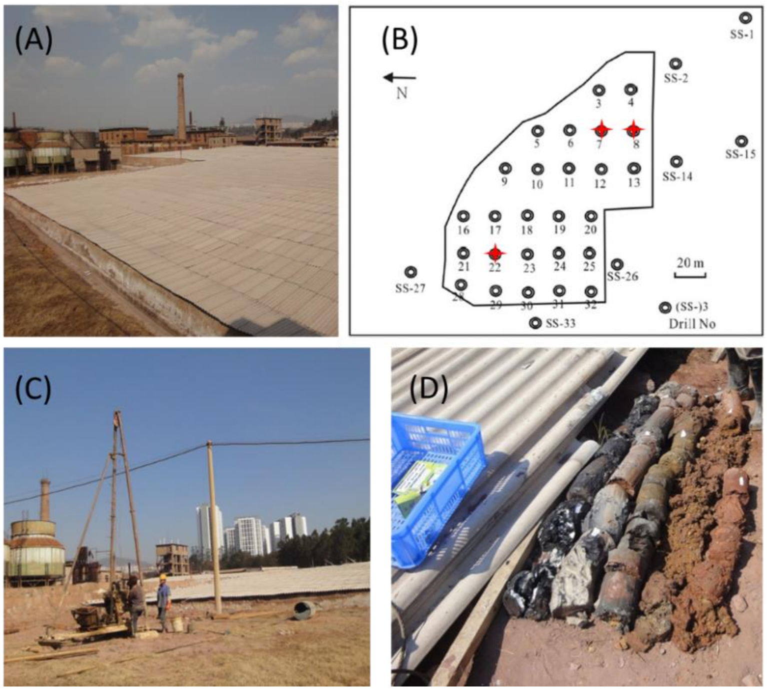

Figure S3. Schematic diagram of the gas exchange chamber (GEC) system set-up for the Hg(0) re-emission experiments.

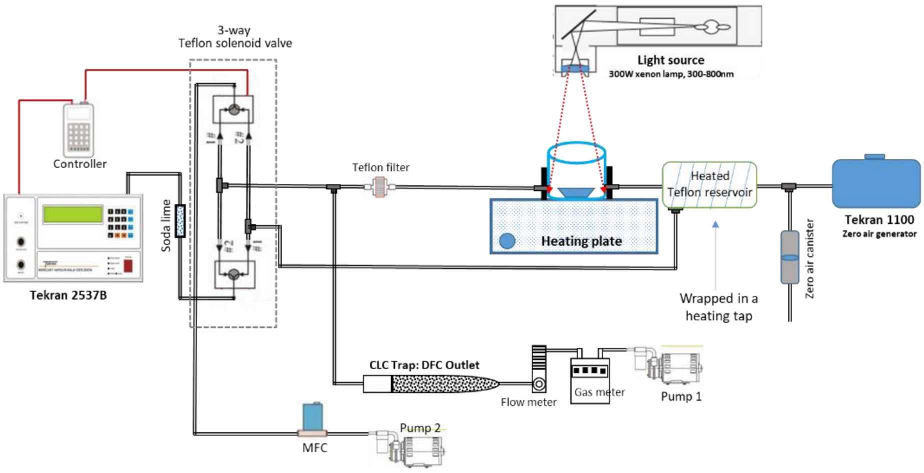

Figure S4. TD-AAS spectra and peak deconvolution of adjacent surface soil AS-5.

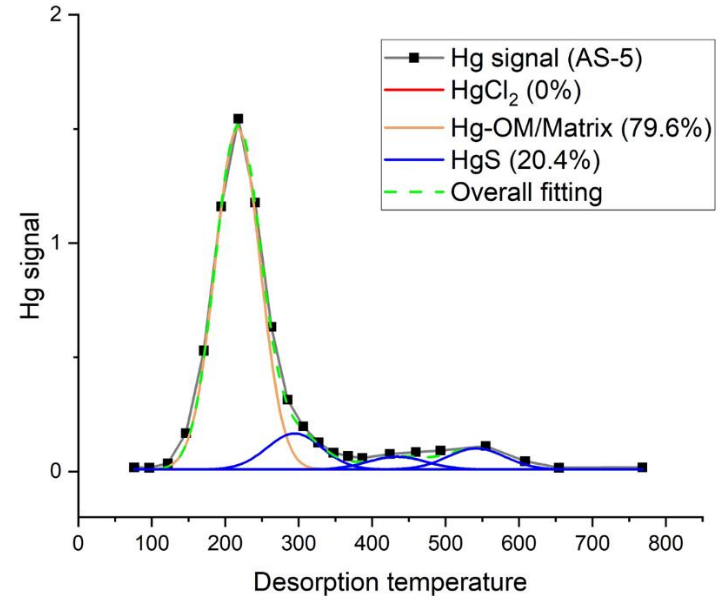

Figure S5. TD-AAS spectra and peak deconvolution of salt-sludge samples collected from the salt-sludge – soil continuum cores: (a) SS7-0, (b) SS22-0, (c) SS22-1.5.

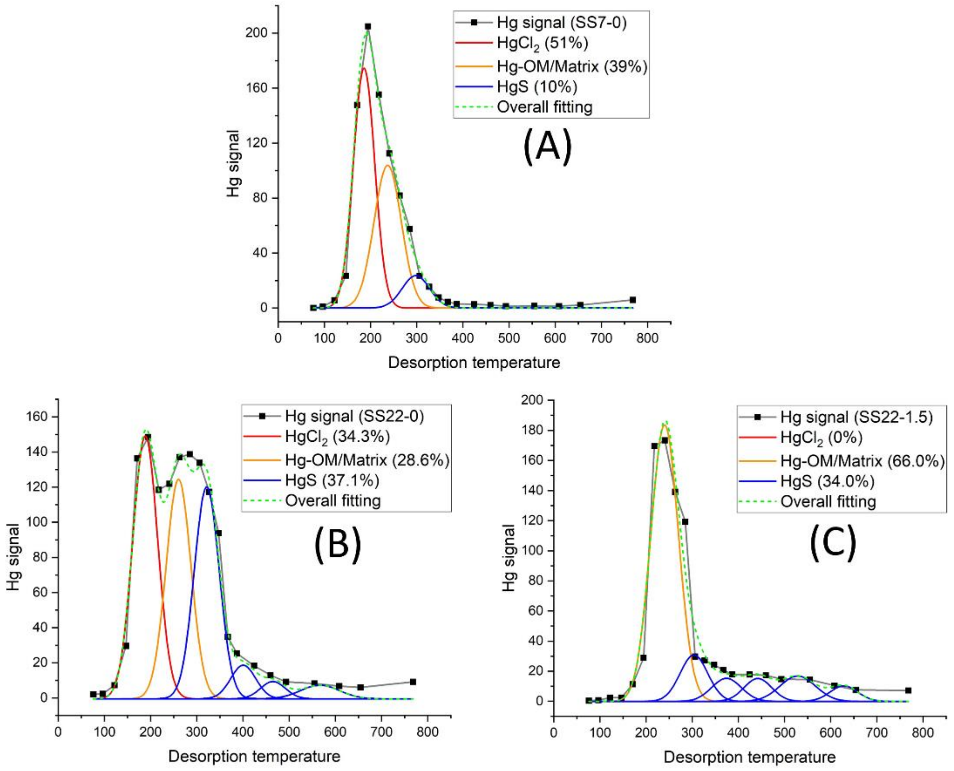

Figure S6. TD-AAS spectra and peak convolution of subsurface soils collected from the salt-sludge – soil continuum cores: (a) SS7-7, (b) SS22-7.

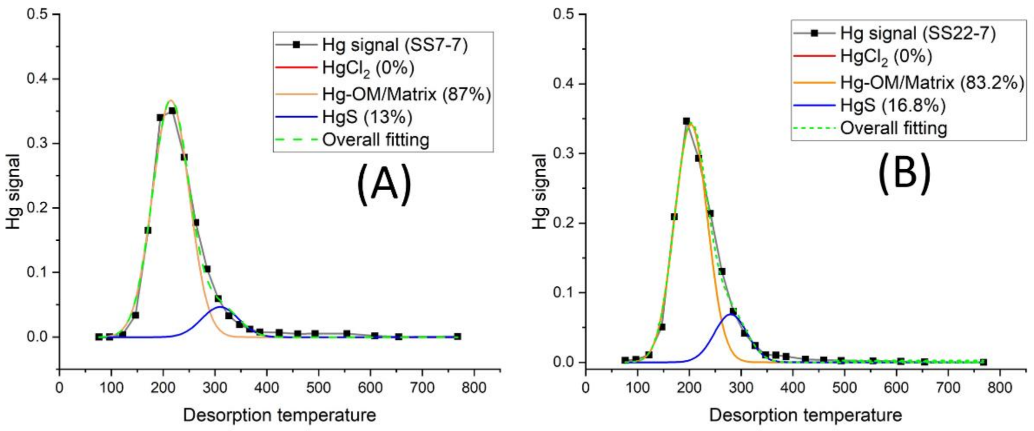

Figure S7. Linear relationship between total Hg (THg) concentrations in salt-sludge – soil cores samples measured in the wet and air-dried samples. Note the THg concentration in wet samples were normalized to dry weight (d.w., mg kg^{-1}) by correcting for gravimetric water content.

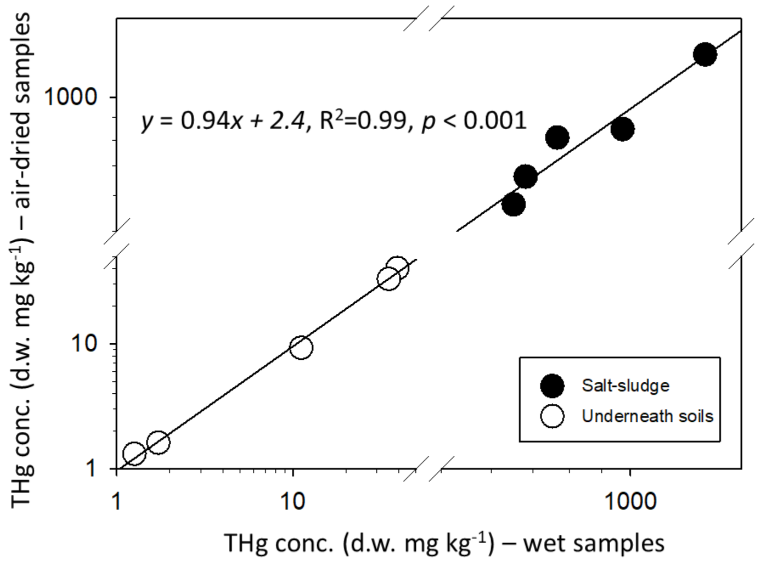

Figure S8. THg concentration in the substrates vs. Hg(0) emission flux. The regression model represent Hg(0) emission flux in response to total Hg concentration in the contaminated adjacent surface soils.

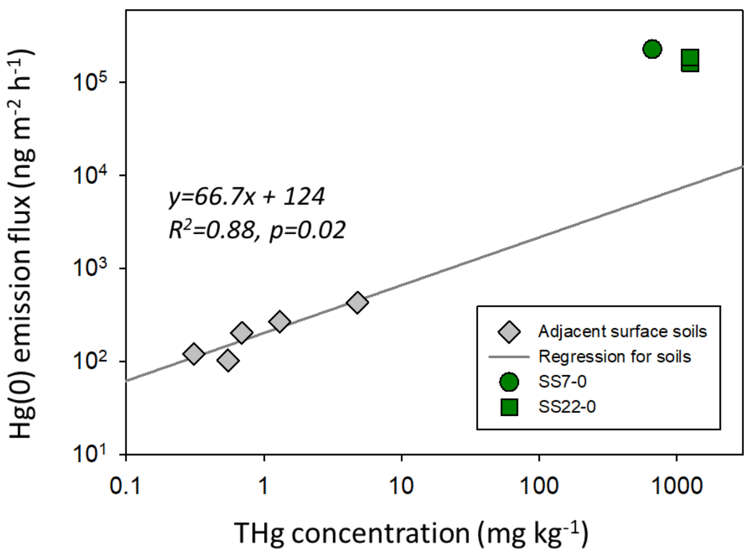

Figure S9. Scatterplots of salt-sludge bulk total Hg and re-emission Hg(0) Δ^{199}Hg vs. Δ^{201}Hg (A), and Δ^{199}Hg vs. δ^{202}Hg (B).

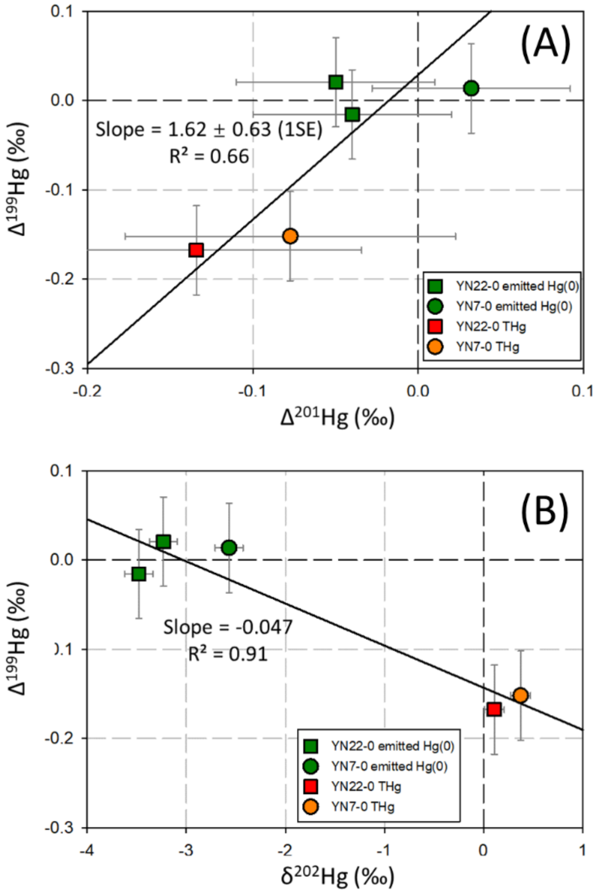

Figure S10. Scatterplots of salt-sludge bulk total Hg Δ^{199}Hg vs. δ^{202}Hg.

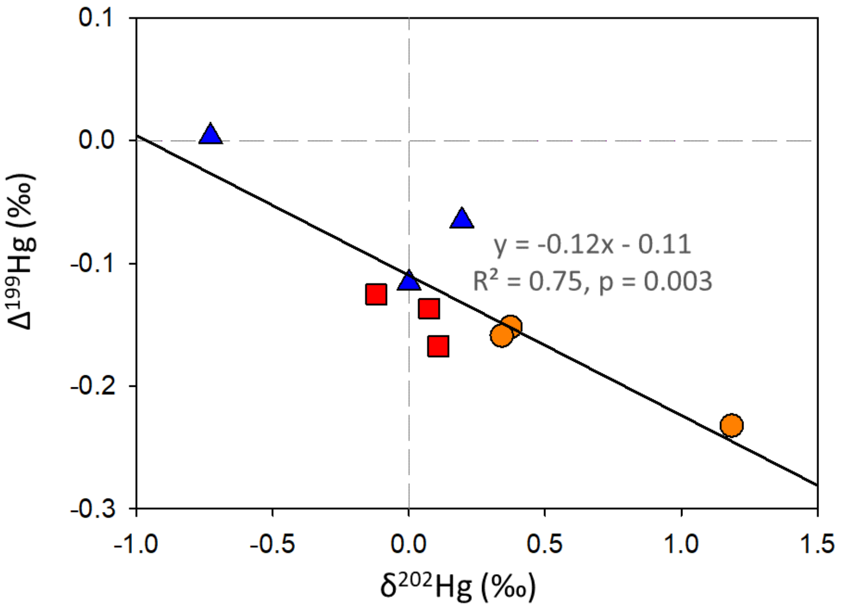

Figure S11. Plots of salt-sludge (color-filled symbols) and subsurface soils (grey-filled symbols) THg δ^{202}Hg vs (A) [THg] and (B) 1/[THg]. A linear relationship was fitted for the subsurface soils in the subfigure B (grey color indicated).

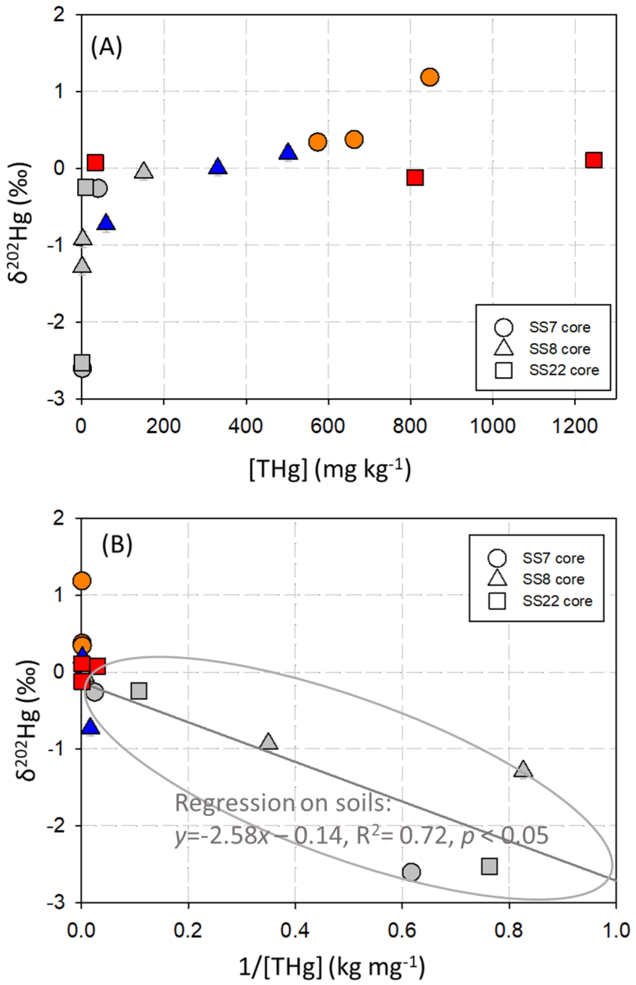

Figure S12. Plots of salt-sludge (color-filled symbols) and subsurface soils (grey-filled symbols) THg Δ^{199}Hg vs (A) [THg] and (B) 1/[THg]. A linear relationship was fitted for the subsurface soils in the subfigure B (grey color indicated).

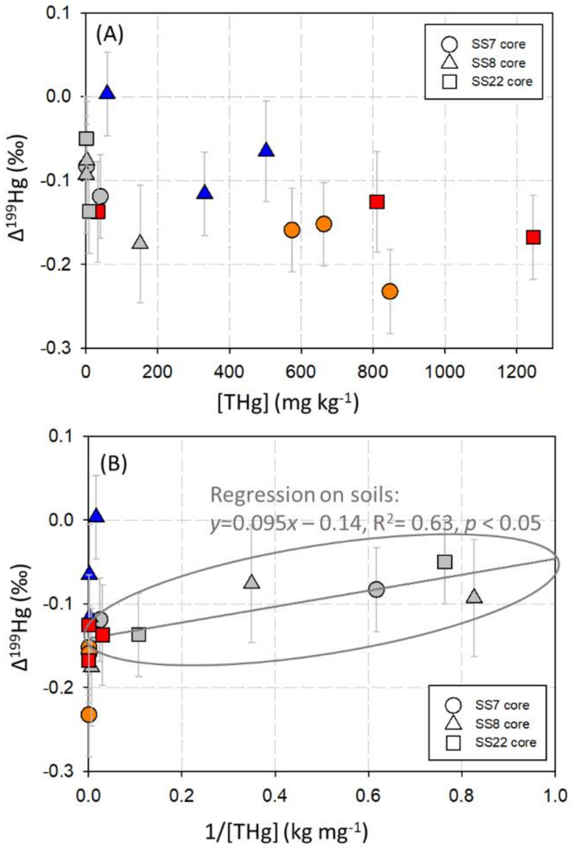

Figure S13. The δ^{202}Hg values enrichment in water-soluble Hg relative to the bulk substrate total Hg (i.e., $\delta^{202}Hg_{water\text{-}soluble}$ - $\delta^{202}Hg_{bulk\ THg}$) along the salt-sludge to soil continuum cores.

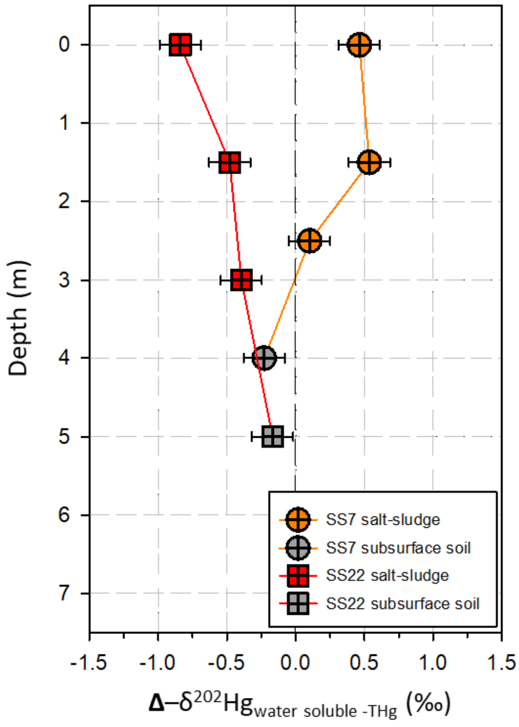

Figure S14. Scatterplots of subsurface soils (underneath the salt-sludge) bulk total Hg Δ^{199}Hg vs. Δ^{201}Hg.

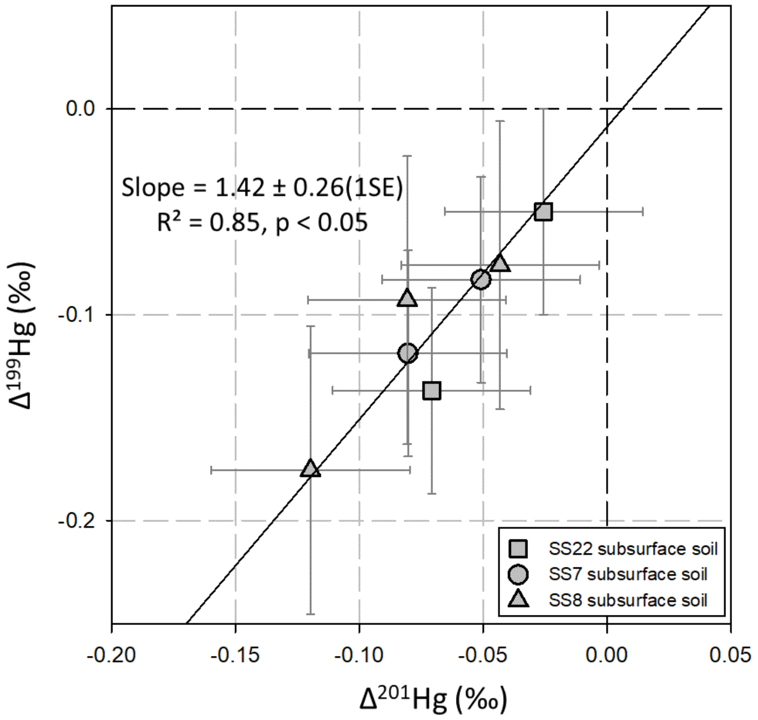

References

1. Zhu, W.; Li, Z.; Li, P.; Yu, B.; Lin, C.-J.; Sommar, J.; Feng, X., Re-emission of legacy mercury from soil adjacent to closed point sources of Hg emission. *Environmental Pollution* **2018,** *242*, 718-727.
2. Bahlmann, E.; Ebinghaus, R.; Ruck, W., Development and application of a laboratory flux measurement system (LFMS) for the investigation of the kinetics of mercury emissions from soils. *J Environ Manage* **2006,** *81*, (2), 114-125.
3. Lin, C. J.; Gustin, M. S.; Singhasuk, P.; Eckley, C.; Miller, M., Empirical models for estimating mercury flux from soils. *Environ. Sci. Technol.* **2010,** *44*, (22), 8522-8528.
4. Gustin, M. S.; Rasmussen, P.; Edwards, G.; Schroeder, W.; Kemp, J., Application of a laboratory gas exchange chamber for assessment of in situ mercury emissions. *Journal of Geophysical Research-Atmospheres* **1999,** *104*, (D17), 21873-21878.
5. Eckley, C. S.; Gustin, M.; Miller, M. B.; Marsik, F., Scaling non-point-source mercury emissions from two active industrial gold mines: influential variables and annual emission estimates. *Environ. Sci. Technol.* **2011,** *45*, (2), 392-399.
6. Gray, J. E.; Pribil, M. J.; Higueras, P. L., Mercury isotope fractionation during ore retorting in the Almadén mining district, Spain. *Chem Geol* **2013,** *357*, 150-157.
7. Sun, R.; Streets, D. G.; Horowitz, H. M.; Amos, H. M.; Liu, G.; Perrot, V.; Toutain, J.-P.; Hintelmann, H.; Sunderland, E. M.; Sonke, J. E., Historical (1850–2010) mercury stable isotope inventory from anthropogenic sources to the atmosphere. *Elementa: Science of the Anthropocene* **2016,** *4*, (1), 000091.
8. Yin, R.; Feng, X.; Wang, J.; Li, P.; Liu, J.; Zhang, Y.; Chen, J.; Zheng, L.; Hu, T., Mercury speciation and mercury isotope fractionation during ore roasting process and their implication to source identification of downstream sediment in the Wanshan mercury mining area, SW China. *Chem Geol* **2013,** *336*, (0), 72-79.
9. Estrade, N.; Carignan, J.; Sonke, J. E.; Donard, O. F. X., Mercury isotope fractionation during liquid-vapor evaporation experiments. *Geochimica Et Cosmochimica Acta* **2009,** *73*, (10), 2693-2711.
10. Ghosh, S.; Schauble, E. A.; Lacrampe Couloume, G.; Blum, J. D.; Bergquist, B. A., Estimation of nuclear volume dependent fractionation of mercury isotopes in equilibrium liquid–vapor evaporation experiments. *Chem Geol* **2013,** *336*, 5-12.
11. Li, P.; Feng, X. B.; Qiu, G. L.; Shang, L. H.; Wang, S. F.; Meng, B., Atmospheric mercury emission from artisanal mercury mining in Guizhou Province, Southwestern China. *Atmospheric Environment* **2009,** *43*, (14), 2247-2251.
12. Wang, C.; Song, Z.; Li, Z.; Zhu, W.; Li, P.; Feng, X., Mercury speciation and mobility in salt slurry and soils from an abandoned chlor-alkali plant, Southwest China. *Science of The Total Environment* **2019,** *652*, 900-906.
